# Mass Spectrometric Analysis of Antibody—Epitope Peptide Complex Dissociation: Theoretical Concept and Practical Procedure of Binding Strength Characterization

**DOI:** 10.3390/molecules25204776

**Published:** 2020-10-17

**Authors:** Bright D. Danquah, Kwabena F. M. Opuni, Claudia Roewer, Cornelia Koy, Michael O. Glocker

**Affiliations:** 1 Proteome Center Rostock, University Medicine Rostock, 18059 Rostock, Germany; danquahbright@yahoo.com (B.D.D.); claudia.roewer@uni-rostock.de (C.R.); cornelia.koy@med.uni-rostock.de (C.K.); 2 School of Pharmacy, University of Ghana, P. O. Box LG53 Legon, Ghana; kfopuni@ug.edu.gh

**Keywords:** mass spectrometric epitope mapping, gas phase immune complex dissociation, apparent gas phase dissociation constants, apparent gas phase activation energies, ITEM-TWO, native mass spectrometry

## Abstract

Electrospray mass spectrometry is applied to determine apparent binding energies and quasi equilibrium dissociation constants of immune complex dissociation reactions in the gas phase. Myoglobin, a natural protein-ligand complex, has been used to develop the procedure which starts from determining mean charge states and normalized and averaged ion intensities. The apparent dissociation constant $K_{D\;m0g}^{\#}=$ 3.60 × 10^−12^ for the gas phase heme dissociation process was calculated from the mass spectrometry data and by subsequent extrapolation to room temperature to mimic collision conditions for neutral and resting myoglobin. Similarly, for RNAse S dissociation at room temperature a $K_{D\;m0g}^{\#}=$ 4.03 × 10^−12^ was determined. The protocol was tested with two immune complexes consisting of epitope peptides and monoclonal antibodies. For the epitope peptide dissociation reaction of the FLAG peptide from the antiFLAG antibody complex an apparent gas phase dissociation constant $K_{D\;m0g}^{\#}=$ 4.04 × 10^−12^ was calculated. Likewise, an apparent $K_{D\;m0g}^{\#}=$ 4.58 × 10^−12^ was calculated for the troponin I epitope peptide—antiTroponin I antibody immune complex dissociation. Electrospray mass spectrometry is a rapid method, which requires small sample amounts for either identification of protein-bound ligands or for determination of the apparent gas phase protein-ligand complex binding strengths.

## 1. Introduction

### 1.1. ESI Mass Spectrometric Analysis of Non-Covalent Complexes

Electrospray mass spectrometric methods have gained broad acceptance for investigation of the constituents of supramolecular complexes and determination of binding surfaces, e.g., for identifying the locations of partial surfaces on antigens which are recognized by an antibody of interest [1]. By contrast, up to now there exists no mass spectrometric method which has gained equal acceptance for investigating gas phase binding strengths of distinct protein-ligand complexes. Previous reports have shown that high pressure mass spectrometry and/or black body irradiation can be applied for analyzing small molecule-ion equilibria and to determine kinetic and thermodynamic properties, such as ion-ligand complex constants in the right order of magnitude [2,3,4] also for small peptides, the protonated glycine dimer being the smallest possible peptide dimer representative [5]. Gas phase dissociation reactions of Leu-enkephaline dimers [6] and of small proteins, such as ubiquitin [7] had been studied as well. Such investigations included the application of the Eyring–Polanyi equation for bimolecular gas phase reactions [8]. Gas phase dissociation profiles under low pressure collision gas conditions have been determined to estimate relative gas phase binding strengths of DNA duplex structures which correlated to the solution phase stabilities [9] and of antibiotic ligand-peptide complexes [10], respectively. Semi-quantitative analysis of glycan ligand-protein binding has been reported as well to estimate binding strengths by ESI-MS [11].

Recently, and still uncommonly, the combination of fast and robust gas-phase epitope mapping methods [12,13] with mass spectrometry-based determination of quasi-thermodynamic information has been published. The latter was obtained based on desolvated and multiply charged and accelerated protein-protein complex ions in the gas phase [14]. These studies, together with reports on collision induced unfolding reactions of protein ions [15], have enabled the development of a method termed ITEM-TWO (Intact Transition Epitope Mapping—Thermodynamic Weak-force Order) [16] that can simultaneously identify epitopes as well as enables to determine gas phase binding strengths of the respective antibody-epitope peptide interactions. Here, in addition to describing all required experimental in-solution handling steps, we introduce the underlying theoretical concept and explain all necessary mathematical calculations in detail through which the apparent dissociation constants and the apparent activation energies of protein-ligand complex dissociation processes in the gas phase are obtained.

### 1.2. Theoretical Concept of Dissociation of Protein-Ligand Complex Ions in the Gas Phase

In a typical Q-ToF instrument, dissociation of protein-ligand complex ions in the gas phase is induced by collisions of multiply charged complex ions with noble gas atoms in a collision chamber at a given collision energy. The ligand dissociation reaction encompasses in its most simple approximation a single transition state, as is indicated by a “one stage” chemical reaction where $k^{\#}$ is the apparent rate constant of product formation (see supplemental information for details).

$$\mathrm{Prot}\times \mathrm{Lig}\rceil^{m^{+}}\;\overset{\;\;\;k\#\;\;\;}{\to }\;\mathrm{Prot}\rceil^{p^{+}}\;+\;\mathrm{Lig}\rceil^{n^{+}}$$

**Figure 1:**
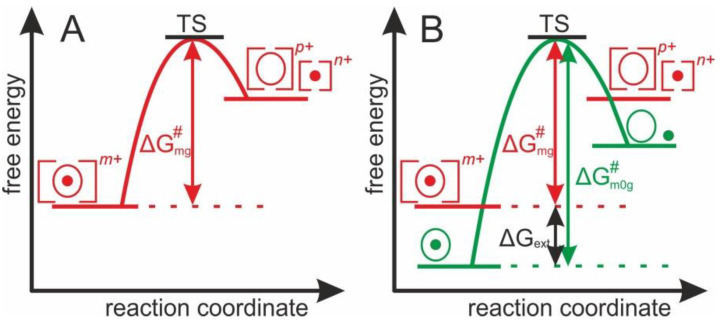
(**A**) Energy diagram showing the apparent Gibbs energy of activation required by charged and accelerated protein-ligand complexes $\left(\Delta G_{mg}^{\#}\right)$ to reach the transition state (**TS**) before dissociating into products ions. (**B**) Energy diagram showing the apparent Gibbs energy of activation required by charged and accelerated protein-ligand complexes ($\Delta G_{mg}^{\#}+\;\Delta G_{ext}^{}$) and by neutral and resting protein-ligand complexes ($\Delta G_{m0g}^{\#})$ to reach the transition state (**TS**) before dissociating into products ions.

To drive the multiply charged protein-ligand complexes’ gas phase dissociation reaction, the apparent Gibbs energy of activation, $\Delta G_{\;mg}^{\#}$ (m: mean of charge states, g: gas phase) is required, as is represented by a “one-stage” energy diagram (Figure 1A). From the transition state (TS), the reaction proceeds irreversibly towards the products. $\Delta G_{\;mg}^{\#}$ is the apparent Gibbs energy of activation of the abundance weighted mean charge state of multiply charged and accelerated protein-ligand complex ions in the gas phase. Yet, after electrospraying there is always an external energy contribution ($\Delta G_{ext}>0$) which needs to be considered during dissociation, as the sum of energies affects the experimentally accessible dissociation energy.

To determine the apparent Gibbs energy of activation of protein complex dissociation of “neutral and resting” protein-ligand complexes, the ESI-dependent external energy contributions need to be considered. Thus, the energy diagram of the complex dissociation reaction requires the introduction of $\Delta G_{m0g}^{\#}$, which is the apparent Gibbs energy of activation that is needed for the dissociation of a protein-ligand complex in the gas phase without external energy contributions (Figure 1B).

### 1.3. ESI Mass Spectral Information Extraction

**Figure 2:**
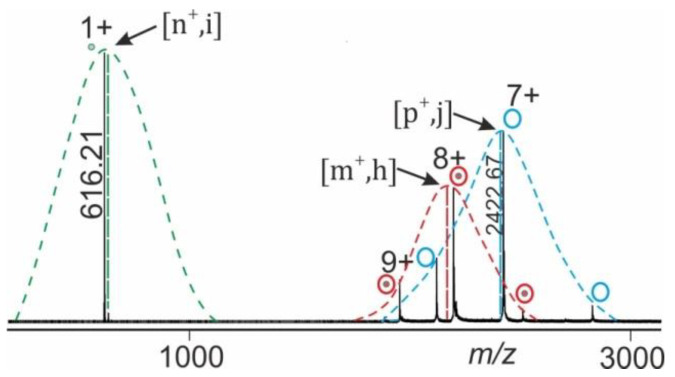
NanoESI mass spectrum of gas phase dissociation of protein complex (e.g., holo-myoglobin; apo-myoglobin—heme complex). Gaussian fits of ion intensities of the related charge state series for each molecular or supra-molecular species (complex) are shown (dashed charge state envelope curves). The arrows point to apices which are determined as maxima of fitted curves. The vertical dashed lines provide heights of charge structure envelopes which represent relative intensities of holo-protein complex ions (h, educts; red), ligand ions (i, product; green), and apo-protein ions (j, product; blue). Locations on the *m/z* axis match with mean charge states of holo-protein complex ions ($m^{+}$, educts), ligand ions ($n^{+}$, product) as well as apo-protein ions ($p^{+}$, product).

ESI-MS of large biomolecules and macromolecular non-covalent complexes in positive ion mode records series of multiply protonated ions which represent a Gaussian ion intensity distribution of individual charge states for a given molecular/supra-molecular species (complex) [17,18]. For semi-quantitative analyses of ESI mass spectra we postulate that the overall ion characteristics, such as gas phase reactivity of complex dissociation, is well represented by the mean charge state ($m^{+}$) of the recorded ion series of a molecular/supra-molecular species (Figure 2).

Mean charge states of each ion species, e.g., holo-protein ions (educts) and of apo-protein as well as of ligand ions (products) can be separately determined from the mass spectrum. Normalization of ion intensities is achieved by summation of all apex values and setting the sum to 100% (equations and calculations are shown in the supplemental information).

### 1.4. Data Analysis Procedure

**Figure 3:**
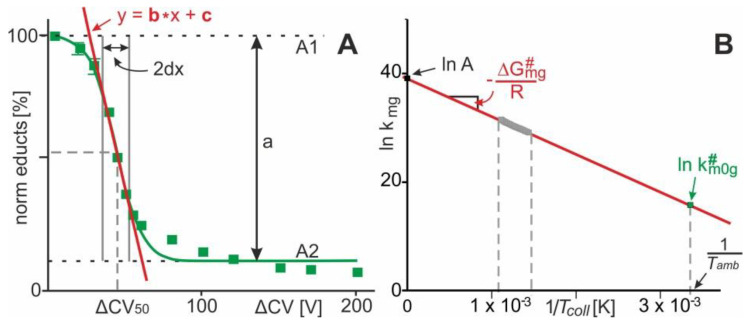
(**A**) Course of normalized ion intensities of complex ions (norm (educts)) as a function of collision cell voltage differences (ΔCV). Each data point is the mean of several independent measurements. Vertical bars give standard deviations. The curve was fitted using a Boltzmann function. The tangent line equation is taken from the Boltzmann fit. “**a**” describes the difference between the lowest and highest data points on the sigmoidal fit. “**2dx**” is the x-axis interval within which the steepest decline of educts is observed; the center of the **2dx** interval is ΔCV**_50_**. (**B**) Arrhenius plot for the course of protein-ligand complex dissociation in the gas phase. The value for $lnk_{m0g}^{\#}$ is taken from the point of the line at $\frac{1}{T_{amb}}$.

A series of mass spectra in which the collision cell voltage difference is increased stepwise records ion signals with varying intensities of all ion species, i.e., educts and products, as they emerge from the collision induced complex dissociation reaction. Plotting the normalized intensities of the educts of the complex dissociation reaction in the gas phase as a function of collision cell voltage difference (ΔCV) provides a sigmoidal shaped curve with Boltzmann characteristics (Figure 3A). The “steep part” of the dissociation reaction dependence (interval 2dx), i.e., the “energy regime” with greatest dependence between educt ion intensities and ΔCV, as well as the determination of $\Delta CV_{50}$ from the Boltzmann fit to the data points is inferred by mathematical procedures which lead to the equation of the tangent line (see supplemental information for calculations).

Since, in the gas phase of a Q-ToF mass spectrometer, the collision of multiply charged and accelerated complex ions takes place upon reaching elevated energies, the collision temperature ($T_{coll}$) that is attained by the complex during collision induced dissociation needs to be considered as well. As proposed by a model for collisional activation [19], $T_{coll}$ can be expressed as the sum of ambient temperature, $T_{amb}$, plus external temperature contribution, $T_{ext}$ (see supplemental information for definitions, energy conversion factors [19,20,21], and equations).

According to the Eyring–Polanyi equation [22], $k^{\#}$ is directly proportional to an apparent thermodynamic quasi equilibrium dissociation constant, $K_{D}^{\#}$. The apparent gas phase thermodynamic quasi equilibrium dissociation constant,$\;K_{D\;mg}^{\#}$, is also given by the relative ion intensities (cf. Figure 1 and Figure 3). Accordingly, for each experimentally set ΔCV value, a corresponding $k_{mg}^{\#}$ value can be calculated (see supplemental information for equations). From the Arrhenius equation, the apparent energy of activation of protein–ligand complex dissociation $\Delta G_{\;mg}^{\#}$ can be determined. Plotting $lnk_{mg}^{\#}$ as a function of $\frac{1}{T_{coll}}$ provides the intercept with the y-axis, which is lnA (pre-exponential factor), and the slope of the line, which is $-\;\frac{\Delta G_{mg}^{\#}}{R}$ (Figure 3B). Note, at $T_{coll}=\;T_{amb}$ = **298 K** it can be concluded that ΔCV = 0. Hence, from the Arrhenius plot a value for $k_{m0g}^{\#}$ is obtained, i.e., the apparent rate constant of dissociation of “neutral and resting” protein-ligand complexes. Similarly, at ΔCV = 0 the value for $K_{D\;m0g}^{\#}$, is calculated, i.e., the apparent gas phase thermodynamic equilibrium dissociation constants of protein-ligand complex dissociation, corrected for external energy contributions; i.e., of “neutral and resting” protein-ligand complexes. Therefore, at ΔCV=0 the value for $\Delta G_{m0g}^{\#}$ is calculated as well, i.e., the apparent Gibbs energy of activation of neutral and resting protein-ligand complexes (see Supplement for equations).

The entire procedure, which is termed “Intact Transition Epitope Mapping—Thermodynamic Weak-force Order (ITEM-TWO)”, starts with either generating the protein-ligand complex by mixing the two components in solution or by simply maintaining the natively obtained protein-ligand complex in an electrospray-compatible solution. No further in-solution sample handling steps are needed. A few microliters of complex-containing solution are loaded into a nano-electrospray capillary and all solubilized components including the protein-ligand complex are simultaneously transferred into the gas phase by electrospray. Mass spectrometric data acquisition involves collision induced dissociation of the complex in the gas phase at various applied collision cell voltage differences (ΔCV). Subsequent in-depth data analysis of intensities of both, resulting product ions and remaining educt ions (survivors) at each of the applied collision energies enables the apparent non-covalent complex stability to be characterized. In this reports supplement, the entire data analysis procedure is described in all detail. In contrast to previous reports the complex dissociation reaction is monitored by investigating the mean charge states and the normalized average intensities of each ion species.

## 2. Results

### 2.1. Procedure Development with Myoglobin and Application to RNAse S Dissociation

**Figure 4:**
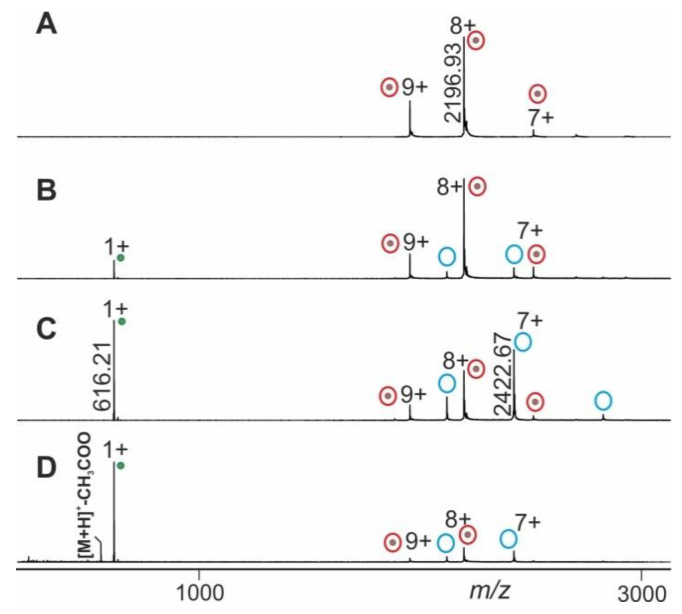
Nano-ESI mass spectra from myoglobin dissociation experiments. Different collision cell voltage differences (ΔCV) are shown. (**A**) 4 V. (**B**) 30 V. (**C**) 60 V. (**D**) 120 V. Charge states and *m/z* values for selected ion signals are given for holo-myoglobin ions (red circles with dots on right ion series), apo-myoglobin ions (blue circles without dots on right ion series) and for the released heme ions (green dots on left ion series). Solvent: 200 mM ammonium acetate, pH 7.

The mass spectral data obtained from electrospraying myoglobin (the holo-myoglobin complex consists of apo-myoglobin plus heme) were collected by following the ITEM-TWO protocol (see Methods section for in-solution handling and data acquisition steps). The ESI mass spectrum of myoglobin (Figure 4) provides an ion series of multiply charged ions from which a mean charge state of 8.1+ and an average mass of 17,566.95 ± 0.46 Da is calculated for holo-myoglobin. After having electrosprayed the myoglobin solution, and upon having switched on the collision gas and having increased the collision cell voltage difference (ΔCV) in a step-wise manner (5–20 V/step), dissociation of holo-myoglobin complexes caused appearance of complex-released heme ions (ligands) in the low mass ranges of the mass spectra (Supplemental Table S1). The mass spectra also showed ion signals of apo-myoglobin (mean charge state: 7.2+) in the high mass ranges (average mass: 16,951.46 ± 0.44 Da) with increasing yields (Figure 4). The *m/z* value of the ion that appeared in the low mass range ([heme]+) was 616.21 and corresponded precisely to the calculated values for singly protonated [heme]+ (*m/z* 616.18), resulting in a mass accuracy of 48 ppm.

Mean charge states of holo-myoglobin ions (m+), apo-myoglobin (n+) and heme (p+) as well as apex heights (holo-myoglobin ions (h, educts), heme ions (i, product), and apo-myoglobin ions (j, product) were extracted (Supplemental Table S2) from the triplicate measurements, averaged and normalized. For each ΔCV setting one spectrum was generated (Figure 4) and analyzed semi-quantitatively by determining Gaussian fits for all molecular/supra-molecular ion species.

After recording mass spectra under increasing collision cell voltage difference settings (ΔCV) and after determining Gaussian fits of charge structures for each ion series, the averaged norm (educts) values were plotted as a function of ΔCV,which resulted in a sigmoidal shaped course that represented the dependence of educt intensities (starting materials) on ΔCV settings (Supplemental Figure S1). All the y-values from the tangent line of the steep decline that fall within the 2dx interval around $\Delta CV_{50}$ are used for the calculation of $ln\;k_{mg}^{\#}$ values which are then used in the Arrhenius plot (Supplemental Figure S2). As shown above at $T_{coll}=\;T_{amb}$ = **298 K** there applies ΔCV = 0 at which the value for $K_{D\;m0g}^{\#}$ is calculated (Table 1). $K_{D\;m0g}^{\#}$ is the apparent gas phase thermodynamic quasi equilibrium dissociation constants of heme loss of “neutral and resting” myoglobin in the gas phase. Then, $\Delta G_{m0g}^{\#}$ is calculated using the van´t Hoff equation (Table 1).

**Figure 5:**
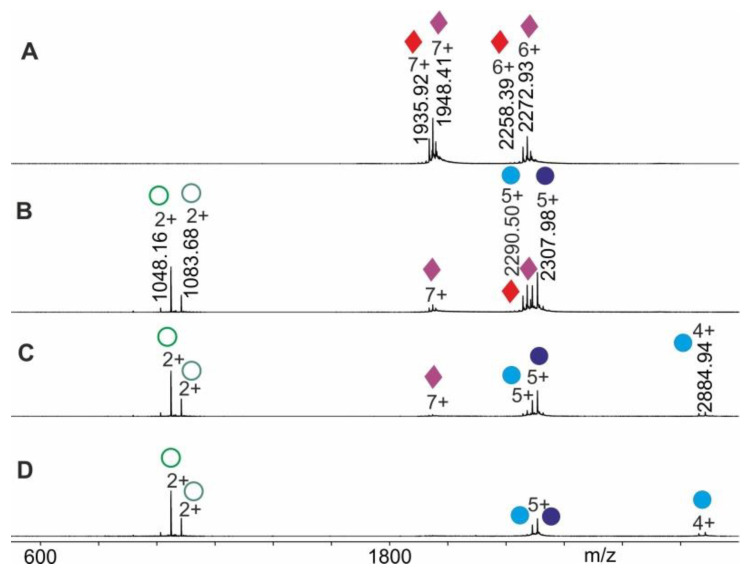
Nano-ESI mass spectra from RNAse S dissociation experiments. Different collision cell voltage differences (ΔCV) are shown. (**A**) 3 V. (**B**) 17 V. (**C**) 30 V. (**D**) 50 V. Charge states and *m/z* values for selected ion signals are given for RNAse S ions (red/purple diamonds on right ion series), S-protein ions (light blue/dark blue filled circles on right ion series) and for the released S-peptide ions (light green/dark green circles on left ion series). Solvent: 200 mM ammonium acetate, pH 7.

The gas phase dissociation reaction of RNAse S was investigated in the same manner. Upon electrospraying RNAse S which had been dissolved in 200 mM ammonium acetate solution, pH 7, the collision cell voltage difference was raised in a step-wise fashion and mass spectra were recorded (see the Methods section for in-solution handling steps). The ESI mass spectrum of RNAse S (Figure 5) provides ion series of multiply charged ions from which mean charge states of 6.4+ and average masses of 13,631.68 ± 0.20 Da and 13,544.23 ± 0.60 are calculated for the two most prominent RNAse S species. Commercial RNAse S represents two prominent protein complexes with clearly differentiated ion series, all of which represent related forms of RNAse S (Supplemental Table S3). For determining the overall apparent activation energy of the S-peptide dissociation reaction from RNAse S, all ion series were considered to equally represent the dissociation process as a whole, meaning that all ion signal intensities were subjected to normalization (Supplemental Table S4).

After recording mass spectra under increasing collision cell voltage difference settings (ΔCV) and after determining Gaussian fits of charge structures for each ion series, the averaged norm (educts) values were plotted as a function of ΔCV, again generating a sigmoidal shaped course (Supplemental Figure S3). As before, all the y-values from the tangent line that fell within the **2dx** interval around $\Delta CV_{50}$ were used for calculating $ln\;k_{mg}^{\#}$ values. These were subjected to draw the respective Arrhenius plot (Supplemental Figure S4). Then, the value for $K_{D\;m0g}^{\#}$ was calculated (Table 1) to represent the apparent gas phase quasi thermodynamic equilibrium dissociation constant of S-peptide loss from “neutral and resting” RNAse S in the gas phase. Finally, $\Delta G_{m0g}^{\#}$ is calculated using the van´t Hoff equation (Table 1).

### 2.2. Application Examples with Epitope Peptide-Antibody Immune Complexes

**Figure 6:**
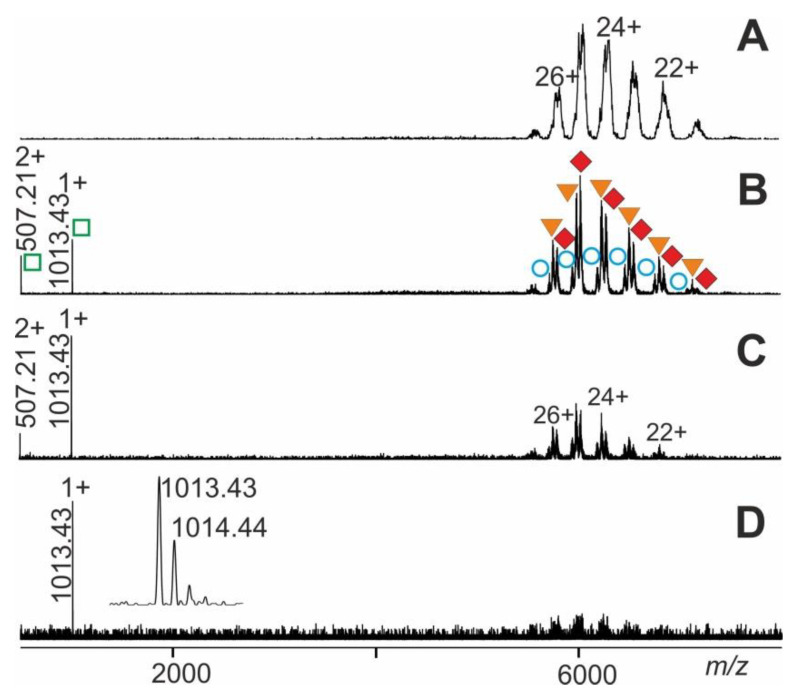
Nano-ESI mass spectra of FLAG-peptide-antiFLAG antibody complex dissociation. Different collision cell voltage differences (ΔCV) are shown. (**A**) 20 V. (**B**) 70 V. (**C**) 120 V. (**D**) 150 V. Charge states and *m/z* values for selected ion signals are given for the immune complexes (antibody plus one FLAG-peptide and antibody plus two FLAG-peptides; filled orange triangles and filled red diamonds, respectively; right ion series), antiFLAG antibody (open blue circles; right ion series), and FLAG-peptide (open green squares; left ion series). The inset shows a zoom of the singly-charged FLAG peptide ion signals. Solvent: 200 mM ammonium acetate, pH 7.

The ITEM-TWO procedure was tested with immune complexes which were generated by mixing an epitope peptide-containing solution with a solution that contained its respective monoclonal antibody. The mixture of antiFLAG antibody with seven peptides was investigated. The mass spectrum of this antibody-peptide mixture showed in the high mass ranges three narrowly spaced multiply charged ion triplets at each charge state (Figure 6). The molecular masses of these triplet ions (charge states from 21+ to 27+; mean charge state 24.6+) were determined to be 148,730 ± 92 Da, 149,799 ± 45 Da, and 150,785 ± 61 Da, which represented the antiFLAG antibody, the antiFLAG antibody with one bound FLAG peptide, and the antiFLAG antibody with two bound FLAG peptides, respectively (Supplemental Table S5). The mass differences between each ion signal triplet provided rather inaccurate mass values for the bound peptide and, therefore, unambiguous identification of the epitope peptide out of the mixture of seven peptides was not possible.

Due to the chosen quadrupole settings, the low mass ions of unbound peptides were filtered out. Yet, upon raising the collision cell voltage difference there appeared isotopically resolved ions in the low *m/z* range of the mass spectrum for the FLAG peptide (see inset in Figure 6) which were recorded with high mass accuracy (20 ppm) and enabled unambiguous identification. It is worth noting that the antiFLAG antibody complex only released the FLAG peptide despite the presence of six other peptides in solution.

By increasing the collision cell voltage differences from 4 V to 200 V in a stepwise manner (20–30 V/step), we observed appearance and incremental rise of doubly and triply charged ion signals in the lower *m/z* range together with gradual disappearance of complex ion signals (Figure 6). These relative complex ion intensities, i.e., the heights of apexes of the Gaussian fits, served as the amounts of the various multiply charged ion series of the antibody-peptide complex ions. The height of the apexes of the Gaussian fits of complex-released epitope peptide ion series were used to represent amounts of the released epitope peptides (Supplemental Tables S5 and S6).

Again, plotting norm (educts) vs. ΔCV, a sigmoidal shaped course was obtained, which represents the dependence of educt intensities on ΔCV (Supplemental Figure S5). Next, the x-axis values (ΔCV) within the intervals dx above and below $\Delta CV_{50}$ were used to determine the corresponding y-axis values using the equation of the tangent line. The resulting y-axis values, i.e., norm (educts), enabled the calculation of $ln\;k_{mg}^{\#}$ values. Plotting increments of $ln\;k_{mg}^{\#}$ vs. $\frac{1}{T_{coll}}$ allowed determination of the part of the apparent dissociation reaction within the “energy regime” located around $\Delta CV_{50}$ (Arrhenius plot; Supplemental Figure S6). In the same manner as was shown above, the calculated value for $k_{m0g}^{\#}$ represented the apparent rate constant of dissociation of “neutral and resting” antibody-epitope peptide complexes. Then, $K_{D\;m0g}^{\#}$ and $\Delta G_{m0g}^{\#}$ (Table 1) were calculated by applying the respective equations.

**Figure 7:**
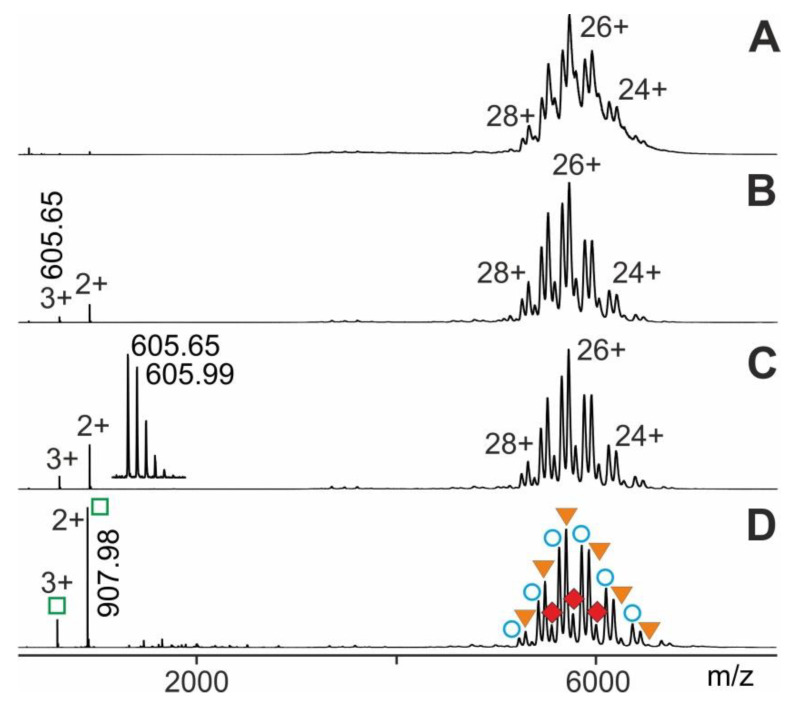
Nano-ESI mass spectra of Tn I-peptide—antiTn I antibody complex dissociation. Different collision cell voltage differences (ΔCV) are shown. (**A**) 4 V. (**B**) 16 V. (**C**) 30 V. (**D**) 80 V. Charge states and *m/z* values for selected ion signals are given for the immune complexes (antibody plus one Tn I-peptide and antibody plus two Tn I-peptides; filled orange triangles and filled red diamonds, respectively; right ion series), antiTn I antibody (open blue circles; right ion series), and Tn I-peptide (open green squares; left ion series). Insert shows a zoom of the triply-charged Troponin I epitope peptide ion signal. Solvent: 200 mM ammonium acetate, pH 7.

At last, we performed ITEM-TWO experiments with an epitope peptide that was derived from human cardiac Troponin I (Tn I) against which was directed a monoclonal antiTroponin I antibody (antiTn I). Generation of the immune complex in solution and subsequent electrospraying of the entire mixture started data acquisition with the respective instrument settings as mentioned (for details see Methods section). The mass spectrum of this antibody-peptide mixture showed in the high mass range three narrowly spaced multiply charged ion triplets at each charge state (Figure 7). The molecular masses of these triplet ions (charge states from 23+ to 28+; mean charge state 25.9+) were determined to be 146,414.75 ± 33 Da, 148,218.75 ± 35 Da, and 150,018.88 ± 33 Da, which were identified to be representing antiTroponin I antibody, antiTroponin I antibody with one bound Troponin I epitope peptide, and antiTroponin I antibody with two bound Troponin I epitope peptides, respectively (Supplemental Tables S7 and S8). Again, upon raising the collision cell voltage difference there appeared isotopically resolved ions in the low *m/z* range of the mass spectrum for the Troponin I epitope peptide (see insert in Figure 7) which were recorded with high mass accuracy (7 ppm).

Again, plotting norm (educts) vs. ΔCV produced a sigmoidal shaped course which represented the dependence of educt intensity on ΔCV following Boltzmann characteristics (Supplemental Figure S7). The y-axis values on the tangent line, i.e., norm (educts), enabled to calculate $ln\;k_{mg}^{\#}$ values. Plotting increments of $ln\;k_{mg}^{\#}$ vs. $\frac{1}{T_{coll}}$ allowed determination of the part of the apparent dissociation reaction within the “energy regime” located around $\Delta CV_{50}$. This Arrhenius plot again provided lnA (pre-exponential factor) as the intercept with the y-axis and $-\;\frac{\Delta G_{mg}^{\#}}{R}$ as the slope of the line (Supplemental Figure S8). In the same manner as described above, the value for $k_{m0g}^{\#}$ was calculated. Similarly, by applying the Eyring-Polanyi equation $K_{D\;m0g}^{\#}$ was determined. Finally, by using the van´t Hoff equation, $\Delta G_{m0g}^{\#}$ was calculated (Table 1) representing binding strengths of “neutral and resting” antibody-epitope peptide complexes.

## 3. Discussion

In solution, the bi-molecular association of the heme group to apo-myoglobin is thought to follow a bimodal process. Association of heme to (partially unfolded) apo-myoglobin is fast (k_A_ ~10^8^ M^−^^1^·s^−^^1^), followed by a slower structural re-arrangement (k ~500 s^−^^1^) to generate natively-folded holo-myoglobin (at pH 7) in which the iron atom of the heme group is then primarily coordinated by His93 [23]. Accordingly, in-solution affinity of at least partially unfolded apo-myoglobin to the heme prosthetic group has been characterized as rather strong and the complex consisting of both components was assumed to possess a K_D_ ~10^−^^11^ M, whereas natively-folded holo-myoglobin has been determined to form an even stronger complex (K_D_ ~10^−^^13^ M to 3 × 10^−^^14^ M) [24,25].

In the gas phase, the stability of heme binding to apo-myoglobin has been studied by dissociating holo-myoglobin in the orifice-skimmer region of an electrospray mass spectrometer, i.e., at high pressure, where complex stability was found to correlate with the activation energy of dissociation of the complex in solution [26,27]. In that investigation, heme dissociation kinetics was studied (i) by spraying solutions with pH 5 and (ii) by looking at selected charge state pairs (e.g., 8+ protonated holo-myoglobin and 7+ charged apo-myoglobin). Activation energies ranged from 73 kJ/mol to 106 kJ/mol, depending on myoglobin amino acid sequence mutations. Similar values of activation energies for heme dissociation have been reported for 9+ protonated holo-myoglobin (92 kJ/mol) and for 10+ protonated holo-myoglobin (85 kJ/mol), respectively [19]. Values from our investigations of holo-myoglobin dissociation are somewhat lower but in general stand in good agreement with reported data. The uncertainty of this method has been estimated to be approx. 10% [14]. Hence, myoglobin is considered to be an adequate standard for developing the ESI-MS method by which protein-ligand dissociation reactions may be studied. Interestingly, from ESI-MS ETD studies of multiply charged myoglobin gas phase ions it was concluded that—depending on the complexes´ charge states—the heme group might be coordinated by one of two histidinyl residues, mainly by His93 but also by His64, or by both, suggesting some similarity between in-solution and gas phase complex structures—at least around the heme binding pocket [25]. The existence of relatively defined macromolecular structures during the heme dissociation process (e.g., as transition state) fits our model, so we postulate that dissociation of immune complexes follows in principle a hard spheres model, i.e., entropy contributions at the transition state are small [14]. In fact, in-solution antibody—antigen interactions are enthalpy-driven [28,29]. Non-covalent forces in the gas phase as well as structural properties of other desolvated protein ions [30,31,32] have demonstrated that higher order protein structures are maintained in the gas phase for a certain period of time [33,34,35] despite absence of solvation [36]. Hence, similar to myoglobin, the decisive structural properties of antibody-antigen complexes seem preserved in the gas phase, at least to some extent.

The rate limiting factor for irreversible dissociation of immune complexes in the gas phase reaction is the activation barrier that needs to be overcome. With an energy input above a critical threshold, immune complex dissociation proceeds irreversibly but comparatively slowly under CID conditions. At each set energy regime, certain portions of immune complexes reach above threshold conditions which results in mixtures of surviving immune complexes and dissociated products within the timeframe of each single measurement. Thus, despite the de facto irreversible character of the dissociation reaction, apparent equilibrium conditions can be assumed. In contrast to previous work we look at average charge states, to represent a respective protein-ligand complex that has been translated into the gas phase by electrospray, and extrapolate to conditions with no additional external energy contributions, such as multiple charging and acceleration of the complex in the gas phase. Both conditions cannot be realized by experiment, because mass spectrometry experiments are performed with accelerated and (multiply) charged ions. Approximation and extrapolation to “resting and neutral” gas phase complexes is expected to provide a better comparison with in-solution data. As we performed all our work using commercial mass spectrometers, we have no means to change the duration times of the ions in the collision cell. However, by keeping all instrument settings (temperature, pressure, charge states of the complexes, gas identity) constant for the entire duration of the experiments (except of the collision cell voltage difference), we assume that reaction times do not differ too much, when comparing dissociation yields and applying the intensities of all ions for our calculations. Consistent with the literature we observed that at higher collision cell voltage differences the dissociation yields were higher as compared to those which were obtained by applying lower collision cell voltage differences.

As a consequence, from all above considerations it appears well possible to semi-quantitatively compare apparent gas phase binding strengths between complexes and to relate these to in-solution dissociation constants of antibody-antigen complexes, after correcting the energy terms, i.e., by subtracting external energy contributions.

## 4. Materials and Methods

### 4.1. Preparation of Myoglobin-Containing Solution

To demonstrate the procedure, electrospray-compatible solutions of myoglobin with neutral pH, in which binding activities are maintained for performing the ITEM-TWO experiments, are prepared. A stock solution was first prepared by dissolving 5.22 mg of myoglobin (lot # 60K7007, Sigma-Aldrich, Steinheim, Germany) in 1 mL of 200 mM ammonium acetate. Next, 50 µL of the stock solution was diluted with 450 µL of 200 mM ammonium acetate. The concentration of the resulting solution was determined to be 0.5 µg/µL using a QubitTM 2.0 Fluorometer (Carlsbad, California, USA). Finally, 100 µL of the 0.5 µg/µL myoglobin solution were further diluted to 200 µL with 200 mM ammonium acetate buffer, pH 7, to obtain a final concentration of 0.25 µg/µL. For each measurement, ca. 3 µL of the 0.25 µg/µL myoglobin solution were loaded into nanoESI capillaries using a microloader pipette tip (Eppendorf, Hamburg, Germany) and were electrosprayed directly.

### 4.2. Preparation of RNAse S-Containing Solution

A stock solution of ca. 1 mg/mL was first prepared by dissolving the lyophilized powder (0.26 mg) of RNAse S (Lot # 52H7034, Sigma-Aldrich, Steinheim, Germany) in 0.26 mL of 200 mM ammonium acetate, pH 7.0. Then, 100 µL were transferred onto a Microcon centrifuge filter with a 3 kDa cutoff (Millipore Corp., Bedford, MA, USA) together with further 200 µL of 200 mM ammonium acetate solution. This solution was centrifuged for 30 min at 13,000 rpm and 23 °C. The eluate was discarded and 200 µL of 200 mM ammonium acetate solution were added onto the filter. This procedure was repeated for three times. Then, the filter was inverted, placed into a new tube, and centrifuged for 5 min at 4500 rpm at 23 °C. A resulting supernatant of approximately 800 µL was collected and the protein concentration was determined to be 0.78 µg/µL using a Qubit^TM^ 2.0 Fluorometer (Carlsbad, CA, USA) assay. For nano electrospray mass spectrometry 2.56 µL of the purified and concentrated RNAse S solution were diluted to a final concentration of 0.2 µg/µL with 7.44 µL of 10% methanol/200 mM ammonium acetate. For each measurement, ca. 3 µL of the RNAse S solution were loaded into nanoESI capillaries using a microloader pipette tip (Eppendorf, Hamburg, Germany).

### 4.3. Preparation of FLAG-Peptide-AntiFLAG Antibody Immune Complex-Containing Solution

A volume of 20 µL of 1 µg/µL of mouse monoclonal antiFLAG M2 antibody (product code F 1804, Sigma-Aldrich, Steinheim, Germany) was first re-buffered into 200 mM ammonium acetate buffer, pH 7, using a centrifugal filter (Amicon Ultra cutoff 50 K; Merck Millipore Ltd., Tullagreen, Carrigtwohill Co Cork, Ireland), as described [10,13]. After buffer exchange, 5 µL of antiFLAG antibody solution (0.2 µg/µL; 1.33 µM) was mixed with 1.5 µL of a peptide mixture of seven peptides containing 10 µM each of GPI peptide (ALKPYSPGGPR, 1141.62 Da), FLAG peptide (DYKDDDDK, 1012.40 Da), Angiotensin II (DRVYIHPF, 1045.53 Da), TRIM21A peptide (LQELEKDEREQLRILGE, 2097.11 Da), TRIM21B peptide (LQPLEKDEREQLRILGE, 2065.12 Da), TRIM21C peptide (LQELEKDEPEQLRILGE, 2038.06 Da), and RA33 peptide (MAARPHSIDGRVVEP-NH2, 1632.86 Da) in a molar ratio of 2.2:1 of peptide to antibody. Solvent for the peptides was 200 mM ammonium acetate buffer, pH 7.

### 4.4. Preparation of Troponin I-Peptide-AntiTn I Antibody Immune Complex-Containing Solution

The human cardiac Troponin I epitope peptide (ENREVGDWRKNIDAL; peptides&elephants, Hennigsdorf, Germany) was obtained as lyophilized powder. The peptide was dissolved in 200 mM ammonium acetate buffer, pH 7, to obtain a peptide concentration of 1.31 µg/µL. The antiTroponin I antibody [MF4] (product code ab38210; Abcam, Cambridge, UK) was obtained dissolved in PBS buffer, pH 7.4. Buffer was exchanged to 200 mM ammonium acetate buffer, pH 7, by loading 21 µL (40 µg) of the antibody stock solution into a centrifugal filter (Microcon with a cutoff of 50 K; Merck Millipore Ltd., Tullagreen, Carrigtwohill, Co. Cork, Ireland). Then 200 mM ammonium acetate buffer, pH 7, were added to reach a volume of 500 µL. The solution was centrifuged at 13,000 rpm for 10 min. The filtrate was discarded. To the retentate on the filter (ca. 30 µL) 470 µL of 200 mM ammonium acetate buffer, pH 7, were added to reach a total volume of 500 µL. The solution was centrifuged again. This centrifugation/re-filling procedure was repeated eight times. After the last spinning, the filter unit was inverted into a new vial and was centrifuged at 4500 rpm for 5 min to collect the retentate (52 µL). Protein concentration was determined to be 0.33 µg/µL using the Qubit^TM^ 2.0 Fluorometer (Carlsbad, CA, USA) assay. To obtain the immune complex with molar ratio of 2.2:1 of peptide to antibody the antiTroponin I antibody solution (0.225 µM) was diluted 1:2 with 200 mM ammonium acetate and 4 µL were mixed with 1.37 µL of the Troponin I peptide solution 1 which previously had been diluted 1:100 with 200 mM ammonium acetate. The immune complex-containing mixture was incubated at room temperature for at least 1 h. For each measurement, 3 µL of antibody-peptide complex-containing solution were loaded into nanoESI capillaries using a microloader pipette tip (Eppendorf, Hamburg, Germany).

### 4.5. Production of NanoESI Capillaries

NanoESI capillaries for offline measurements were prepared in-house from borosilicate glass tubes of 1 mm outer and 0.5 mm inner diameters (BF 100-50-10, Sutter Instruments, Novato, CA, USA), using a P-1000 Flaming/BrownTM micropipette puller system (Sutter Instruments, Novato, CA, USA). Capillaries were gold-coated using a sputter coater BalTec SCD 0045 (Bal-Tech, Balzers, Liechtenstein) with the following conditions: current was set to 20 mA for 150 s, table distance was 5 cm, while vacuum was ca. 10^−3^ mbar and Argon gas pressure maintained at ca. 10^−2^ mbar [16].

### 4.6. Q-ToF 2 Instrument Settings and Data Aquisition

Nano-ESI-MS measurements were performed using a Q-TOF 2 instrument (Waters MS-Technologies, Manchester, UK). The pressure in the source region of the mass spectrometer was manually adjusted to 2.24 mbar using the speedy valve [16]. ITEM-TWO measurements were performed with the following instrumental settings: source temperature, 50 °C; capillary voltage, 1.3 kV; sample cone voltage, 30 V; extractor cone voltage, 3 V; collision voltage, 4 V; pusher time, 124 µs. The Quadrupole and ToF analyzer pressures were typically between ca. 2.0 × 10^−5^ mbar and 2.50 × 10^−7^ mbar, respectively. All mass spectra were acquired in positive-ion mode with a mass window of *m/z* 200–4000. The *m/z* axis was calibrated using 50% TFE in 1% orthophosphoric acid. The collision gas was then switched on (1.25 bar) and collision cell voltage differences were increased in a stepwise manner (5–20 V/step) to cause dissociation of the complexes. At each collision cell voltage difference setting, a mass spectrum was recorded for 2 min, each. All scans for a given collision cell voltage difference were combined to generate an average spectrum. Q-ToF MS data were acquired and minimally processed using the MassLynx software version 4.0 (Waters MS-Technologies, Manchester, UK). From each spectrum, the ion intensities (in arbitrary units) were deduced [16].

### 4.7. Synapt G2S Instrument Settings and Data Aquisition

Nano-ESI-MS measurements were performed using a Synapt G2S instrument (Waters MS-Technologies, Manchester, UK). ITEM-TWO measurements of the Troponin I peptide—antiTroponin I complex were performed with the following instrumental settings: source temperature, 50 °C; capillary voltage, 1.8 kV; sample cone voltage, 110 V; source offset voltage, 110 V; trap gas flow, 8.0 mL/min; cone gas flow, 100 L/h. All mass spectra were acquired in positive-ion mode applying a mass window of *m/z* 200–8000. The *m/z* axis was calibrated using 1 mg/mL sodium iodide dissolved in an isopropanol/water solution (50:50, *v*/*v*). The quadrupole analyzer was used to block transmission of lower molecular weight ions: M1 = 4000 with dwell time of 25% and ramp time of 25%; M2 = 5000 with dwell time of 25% and ramp time of 25%; M3 = 6000. The surviving antibody-peptide complexes were dissociated in the first collision cell (TRAP) by increasing the collision cell voltage difference in a stepwise manner (2–10 V/step). Data were acquired and processed with MassLynx software version 4.1 (Waters MS-Technologies, Manchester, UK). At each collision cell voltage difference setting a mass spectrum was recorded for 1 min, each. All scans for a given collision cell voltage difference were combined to generate an average spectrum. From each spectrum the ion intensities (in arbitrary units) were deduced.

### 4.8. Mass Spectral Analysis

The Savitzky–Golay method was used for smoothing in five cycles with a window of 10 for the high mass range and in three cycles with a window of 5 for the low mass range, respectively. Fractions of educts and products were derived from heights of ion signals of complex (educt) and its dissociated constituents (products) at all applied collision cell voltage differences (ΔCV). At each applied ΔCV setting the height of apex of Gaussian fit of the multiply charged ion series of the respective complex as well as of its constituents was determined by recording the intensities and respective *m/z* values of all ion signals for each charge state. Next, these ion intensities were plotted against their respective *m/z* values and fitted to a Gaussian curve (Figure 2) using Origin version 8.1 (OriginLab Corporation, Northampton, MA, USA). Intensity apexes of each molecular species or complex from the heights of the individual ion signals were determined as well. The relative amounts of products, f(products), and relative amounts of educts, f(educts), from the nanoESI mass spectra at given ΔCV settings were determined. The plots of normalized educt intensities i.e., norm(educts) vs. ΔCV were fitted to a Boltzmann curve (R^2^ ≥ 0.99).

The mass spectrometry data have been deposited to the ProteomeXchange Consortium via the PRIDE [37] partner repository with the dataset identifier PXD021296.

## 5. Conclusions

ITEM-TWO is able (i) to determine epitopes and (ii) to investigate the epitopes´ binding strengths in the gas phase. Mixing of antigen or epitope peptide and antibody solutions is the only required in-solution handling step when the complex components are dissolved in electrospray-compatible solutions. From normalized ion intensities, the apparent gas phase quasi equilibrium dissociation constants ($K_{D\;m0g}^{\#}$) can be deduced from which apparent dissociation activation energies for neutral and resting immune complexes in the gas phase ($\Delta G_{\;mOg}^{\#}$) can be calculated. As suitable electrospray mass spectrometry equipment has become amply available, our ITEM-TWO method should be easily adaptable by mass spectrometry laboratories all around the world.

## Figures and Tables

**Table 1:** Apparent kinetic and apparent quasi-thermodynamic values for gas phase dissociation of non-covalent complexes.

Complex ^(a)^	Mean Charge ± std. dev. ^(b)^	∆CV_50_ (V)	dx (V)	$k_{m0g}^{\#}$ (1/s)	$K_{D\;m0g}^{\#}$ (Ø) ^(b)^	$\Delta G_{m0g}^{\#}$ (kJ/mol)
myoglobin	8.1 ± 0.01	44.0	8.5	5.1 × 10 ^9^	3.60 × 10^−12^	65.3
RNAse S	6.4 ± 0.20	12.5	4.6	7.3 × 10 ^10^	4.03 × 10^−12^	65.0
FLAG	24.6 ± 0.30	89.0	19.8	7.9 × 10 ^10^	4.04 × 10^−12^	65.0
troponin I	25.9 ± 0.14	26.4	15.9	2.2 × 10 ^12^	4.58 × 10^−12^	64.7

(a) “neutral and resting” complex; (b) unitless number.

## Supplementary Materials

The following are available online, Equations and theoretical explanations. Figure S1: Course of normalized ion intensities of holo-myoglobin ions (*norm* (*educts*)) as a function of collision cell voltage differences (ΔCV), Figure S2: Arrhenius plot for the course of myoglobin dissociation in the gas phase, Figure S3: Course of normalized ion intensities of RNAse S ions (*norm* (*educts*)) as a function of collision cell voltage differences (ΔCV), Figure S4: Arrhenius plot for the course of RNAse S dissociation in the gas phase, Figure S5: Course of normalized ion intensities of FLAG-peptide—antiFLAG antibody ions (*norm* (*educts*)) as a function of collision cell voltage differences (ΔCV), Figure S6: Arrhenius plot for the course of FLAG-peptide—antiFLAG antibody complex dissociation in the gas phase, Figure S7: Course of normalized ion intensities of Troponin I peptide—antiTroponin I antibody complex (*norm* (*educts*)) as a function of collision cell voltage differences (ΔCV), Figure S8: Arrhenius plot for the course of Troponin I peptide—antiTroponin I antibody complex dissociation in the gas phase, Table S1: Ion intensities, charge states, and m/z values for myoglobin at various collision cell voltage difference settings, Table S2: Apex heights and mean charge states of educt and product ion signals upon gas phase dissociation of myoglobin, Table S3: Ion intensities, charge states, and m/z values for RNAse S at various collision cell voltage difference settings, Table S4: Apex heights and mean charge states of educt and product ion signals upon gas phase dissociation of RNAse S, Table S5: Ion intensities, charge states, and m/z values for FLAG-peptide—antiFLAG antibody complex at various collision cell voltage difference settings, Table S6: Apex heights and mean charge states of educt and product ion signals upon gas phase dissociation of FLAG-peptide—antiFLAG antibody complex, Table S7: Ion intensities, charge states, and m/z values for TroponinI peptide—antiTroponinI at various collision cell voltage difference settings, Table S8: Apex heights and mean charge states of educt and product ion signals upon gas phase dissociation of TroponinI immune complex.
