# Supplementary material for: Mass Spectrometric Analysis of Antibody—Epitope Peptide Complex Dissociation: Theoretical Concept and Practical Procedure of Binding Strength Characterization
Source: Molecules. 2020 Oct 17;25(20):4776. doi: 10.3390/molecules25204776 (PMC7587528; doi:10.3390/molecules25204776)
Supplement: Supplementary file 1 [file molecules-25-04776-s001.pdf]

molecules

Article

Mass Spectrometric Analysis of Antibody – Epitope Peptide Complex Dissociation: Theoretical Concept and Practical Procedure of Binding Strength Characterization

Bright D. Danquah [1], Kwabena F.M. Opuni [2], Claudia Röwer [1], Cornelia Koy [1], and Michael O. Glocker [1,*]

[1] Proteome Center Rostock, University Medicine Rostock, Rostock, Germany; michael.glocker@uni-rostock.de
[2] School of Pharmacy, University of Ghana, Legon, Ghana; kfopuni@ug.edu.gh
* Correspondence: michael.glocker@uni-rostock.de; Tel.: (+49 - 381 - 494 4930)

Supplement

Equations

Mean charge states of each ion species, *i.e.* educts (e.g. holo-myoglobin ions) and of products (e.g. apo-myoglobin) as well as of ligands (e.g. heme ions) are obtained from the mass spectrum at a given collision cell voltage difference (**ΔCV**) instrument setting (cf. Figure 2) and can be separately determined according to equation (1).

$$\mathbf{m^+} = \sum\left[\mathbf{z^i} * \left(\frac{\mathbf{h_y^i}}{\sum \mathbf{h_y}}\right)\right] \quad (1)$$

where

$\mathbf{m^+}$ abundance weighted mean charge state of the multiply charged ion series
$\mathbf{z^i}$ individual charge state from the multiply charged ion series
$\mathbf{h_y^i}$ individual ion intensity of the ion with charge state $\mathbf{z^i}$ from the multiply charged ion series
$\sum \mathbf{h_y}$ sum of the intensities of all the multiply charged ions from the multiply charged ion series

Heights of Gaussian fit apices of ion signals represent relative intensities or fractions of both, products and educts, where $\mathbf{h}$ is the height of apex of the multiply charged complex ions (e.g. holo-myoglobin; starting material), $\mathbf{i}$ is the height of apex of ligand ions (e.g. heme), and $\mathbf{j}$ is the height of apex of the multiply charged protein ions (e.g. apo-myoglobin). Normalization of ion intensities is achieved by summation of all apex values and setting the sum to 100 %. In cases where only one or few charge states of a ligand / protein / complex appeared in the spectrum, more data points for fitting a Gaussian curve have been added by defining other charge states higher and lower than those of the appearing ion signals with respective *m*/*z* values and setting them to have "0" intensities.

$$\mathbf{h + i + j} \triangleq \mathbf{100\ \%} \quad (2)$$

$$\mathbf{norm\ (products)} = \frac{\mathbf{i+j}}{\mathbf{h+i+j}} \times \mathbf{100\%} \quad (3)$$

or:

$$\mathbf{norm\ (educts)} = \frac{\mathbf{h}}{\mathbf{h+i+j}} \times \mathbf{100\%} \quad (4)$$

$$\mathbf{norm\ (educts)} = \mathbf{100\%} - \mathbf{norm\ (products)} \quad (5)$$

Plotting the normalized intensities of the educts of the complex dissociation reaction in the gas phase as a function of collision cell voltage difference (**ΔCV**) provides a sigmoidal shaped curve with Boltzmann characteristics (cf. Figure 3A). The "steep part" of the dissociation reaction dependence (interval 2dx), *i.e.* the "energy regime" with greatest dependence between educt ion intensity changes and **ΔCV**, as well as the determination of $\mathbf{\Delta CV_{50}}$ from the Boltzmann fit to the data points is inferred. Also, from the Boltzmann curve, the equation of the tangent line is deduced.

$$y = b \bullet x + c \quad (6)$$

Slope of tangent line:

$$b = \frac{A2 - A1}{4dx} \quad (7)$$

Intercept of the tangent line with the y – axis:

$$c = \frac{A2 + A1}{2} - \frac{A2 - A1}{4dx} \bullet |\Delta CV_{50}| \quad (8)$$

Once slope and intercept are obtained, the equation of the tangent line is defined as:

$$y = \frac{A2 - A1}{4dx} x + \frac{A2 + A1}{2} - \frac{A2 - A1}{4dx} \bullet |\Delta CV_{50}| \quad (9)$$

With respect to determine apparent activation energies for dissociation of protein complexes formation of an excited intermediate is assumed. The dissociation reaction follows equation (10).

$$\boldsymbol{Prot \bullet Lig]^{m^+}} \underset{k_{-1}}{\overset{k_1}{\rightleftharpoons}} [\boldsymbol{Prot \bullet Lig}]^{\ddagger}]^{m^+} \xrightarrow{k_2} \boldsymbol{Prot]^{p^+}} + \boldsymbol{Lig]^{n^+}}$$

where

$\boldsymbol{Prot \bullet Lig]^{m^+}}$ multiply charged protein - ligand complex ions

$[\boldsymbol{Prot \bullet Lig}]^{\ddagger}]^{m^+}$ excited intermediate of multiply charged protein - ligand complex ions

$\boldsymbol{Lig]^{n^+}}$ singly / multiply charged ligand ions

$\boldsymbol{Prot]^{p^+}}$ multiply charged protein ions

$\boldsymbol{m^+}$ abundance weighted mean charge state of protein - ligand complex ions

$\boldsymbol{n^+}$ abundance weighted mean charge state of ligand ions

$\boldsymbol{p^+}$ abundance weighted mean charge state of protein ions

$\boldsymbol{k_1}$, $\boldsymbol{k_{-1}}$, $\boldsymbol{k_2}$ rate constants of partial reactions

The apparent rate of product formation, $\boldsymbol{k^{\#}}$, becomes independent of $\boldsymbol{k_2}$ and, hence, of an excited intermediate, when formation of a transition state (TS) is postulated, *i.e.* under conditions where:

$$\mathbf{k_2} \gg \mathbf{k_1} > \mathbf{k_{-1}}$$

Then, equation (10) can be abbreviated to:

$$\mathbf{Prot \bullet Lig]^{m^+}} \xrightarrow{\mathbf{k^{\#}}} \mathbf{Prot]^{p^+}} + \mathbf{Lig]^{n^+}}$$

where

$\boldsymbol{k^{\#}}$ apparent rate constant of product formation.

The energy diagram of the complex dissociation reaction describes the experimental situation (cf. Figure 1A).

Since by electrospray there is always an external energy contribution ($\Delta\mathbf{G_{ext}}$) which needs to be considered, the sum of energies affects the experimentally accessible dissociation activation energy when analyzing dissociation of electrosprayed complex ions.

$$\Delta\mathbf{G_{ext}} > 0 \quad (13)$$

$$\Delta\mathbf{G_{ext}} = \Delta\mathbf{G_{CR}} + \Delta\mathbf{G_{kin}} \quad (14)$$

where

$\Delta\boldsymbol{G_{ext}}$ external energy of the protein - ligand complex ions due to multiple charging and acceleration in the gas phase,

$\Delta\boldsymbol{G_{CR}}$ energy contribution from charge repulsion in multiply charged protein – ligand complex ions in the gas phase,

$\Delta\boldsymbol{G_{kin}}$ kinetic energy of protein - ligand complex ions in the gas phase.

$\Delta\boldsymbol{G_{kin}}$ includes the energy contribution arising from the applied collision cell voltage difference ($\Delta\boldsymbol{CV}$) in the collision cell and $\Delta\boldsymbol{G_{CR}}$ arises from charge repulsion.

Similarly, because in the gas phase of a Q-ToF mass spectrometer, collision of multiply charged and accelerated complex ions takes place upon reaching elevated energies, the collision temperature ($\mathbf{T_{coll}}$) which is attained by the complex during collision induced dissociation needs to be considered as well. As proposed by a model for collisional activation, [15] $\mathbf{T_{coll}}$ can be expressed as:

$$\mathbf{T_{coll}} = \mathbf{T_{amb}} + \mathbf{T_{ext}} \quad (15)$$

where

$\mathbf{T_{coll}}$ protein-ligand temperature during collision in the collision cell

$\mathbf{T_{amb}}$ absolute ambient temperature, 298 K

$\mathbf{T_{ext}}$ temperature increment caused by acceleration and multiple charging of ions

and

$$\mathbf{T_{ext}} = \frac{\mathbf{x\,m\,e\,\Delta CV}}{\mathbf{3N_{at}\,k_B}} \quad (16)$$

where

x overall kinetic energy converted to internal energy, equals 0.24 [15]

m abundance weighted mean charge state of multiply charged and accelerated protein - ligand complex ions

e fundamental charge, 1.602176634 $x10^{-19}$ C

$\mathbf{\Delta CV}$ collision cell voltage difference

$\mathbf{N_{at}}$ number of atoms in the protein - ligand complex

$\mathbf{k_B}$ Boltzmann constant, $1.38064852x10^{-23}$ J/K

Substituting equation (16) into equation (15) yields:

$$\mathbf{T_{coll}} = \mathbf{T_{amb}} + \frac{\mathbf{x\,m\,e}}{\mathbf{3N_{at}\,k_B}} * \mathbf{\Delta CV} \quad (17)$$

where:

$\mathbf{T_{coll}}$ protein-ligand temperature during collision in the collision cell

$\mathbf{T_{amb}}$ absolute ambient temperature, 298 K

x overall kinetic energy converted to internal energy, x = 0.24 [15]

m abundance weighted mean charge state of multiply charged and accelerated protein - ligand complex ions

e fundamental charge, 1.602176634 $x10^{-19}$ C

$\mathbf{N_{at}}$ number of atoms in the protein - ligand complex

$\mathbf{k_B}$ Boltzmann constant, $1.38064852x10^{-23}$ J/K

$\mathbf{\Delta CV}$ collision cell voltage difference

To determine the apparent Gibbs energy of activation of protein complex dissociation of "neutral and resting" protein - ligand complexes, the ESI-dependent external energy contributions needs to be considered. The conditions for "neutral and resting" protein - ligand complexes are met when:

$$\mathbf{\Delta G_{ext}} = \mathbf{0} \quad (18)$$

It can be concluded that condition $\Delta\boldsymbol{G_{ext}} = \mathbf{0}$ are met at:

$$\mathbf{T_{ext}} = \mathbf{0} \quad (19)$$

and therefore equation (15) is reduced to:

$$\mathbf{T_{coll}} = \mathbf{T_{amb}} \quad (20)$$

which sets the temperature of the dissociation reaction of "neutral and resting" complexes to:

$$\mathbf{T_{coll}} = \mathbf{T_{amb}} = 298\ \mathrm{K} \tag{21}$$

Considering dissociation of "neutral and resting" protein complex ions, i.e. "$\Delta \boldsymbol{G_{ext}} = \mathbf{0}$" conditions, the energy diagram of the complex dissociation reaction requires the introduction of $\boldsymbol{\Delta G^{\#}_{m0g}}$, which is the apparent Gibbs energy of activation that is needed for the dissociation of a protein - ligand complex in the gas phase without external energy contributions (cf. Figure 1B).

$\Delta \boldsymbol{G^{\#}_{m0g}}$ is the apparent Gibbs energy of activation of neutral and resting protein - ligand complexes in the gas phase derived from multiply protonated and accelerated protein - ligand complexes (m: mean of charge states, 0: no additional external energy, g: gas phase).

$$\Delta \mathbf{G^{\#}_{m0g}} = \Delta \mathbf{G^{\#}_{mg}} + \Delta \mathbf{G_{ext}} \tag{22}$$

where

m mean of charge states, is "0" in case of "neutral and resting" complex,

0 without external energy contributions,

g gas phase.

According to the Eyring-Polanyi equation [19], $\mathbf{k^{\#}}$ is directly proportional to an apparent thermodynamic quasi equilibrium dissociation constant, $\mathbf{K^{\#}_{D}}$.

$$\mathbf{k^{\#}} = \frac{\boldsymbol{\kappa}\, \mathbf{k_B\, T}}{\mathbf{h}} * \mathbf{K^{\#}_{D}} \tag{23}$$

where

$\mathbf{k^{\#}}$ apparent rate constant of total reaction,

$\boldsymbol{\kappa}$ transmission coefficient; is equal to 1 as the products do not re-cross to the transition state,

$\mathbf{k_B}$ Boltzmann constant, 1.38064852 x 10^{-23} J/K,

T absolute temperature, K,

h Planck's constant, 6.62607015 x 10^{-34} Js,

$\mathbf{K^{\#}_{D}}$ apparent thermodynamic quasi equilibrium dissociation constant.

Regarding mean charge states of complex ions and of gas phase collision temperatures, equation (23) is transformed to:

$$\mathbf{k^{\#}_{mg}} = \frac{\boldsymbol{\kappa}\, \mathbf{k_B\, T_{coll}}}{\mathbf{h}} * \mathbf{K^{\#}_{D\ mg}} \tag{24}$$

where:

$\mathbf{k^{\#}_{mg}}$ apparent rate constant of dissociation reaction of the mean charge state of multiply charged and accelerated protein - ligand complex ions in the gas phase

$\mathbf{T_{coll}}$ protein – ligand complex temperature during collisional dissociation in the collision cell

m abundance weighted mean charge state of multiply charged and accelerated protein - ligand complex ions

g gas phase

$\mathbf{K^{\#}_{D\ mg}}$ apparent gas phase thermodynamic equilibrium dissociation constants of protein - ligand complex dissociation

The apparent gas phase thermodynamic quasi equilibrium dissociation constant, $\boldsymbol{K^{\#}_{D\ mg}}$, is also given by:

$$\mathbf{K^{\#}_{D\ mg}} = \frac{\mathbf{f(products)}}{\mathbf{f(educts)}} \tag{25}$$

combining equations (24) and (25) yields:

$$\mathbf{k^{\#}_{mg}} = \frac{\boldsymbol{\kappa}\, \mathbf{k_B\, T_{coll}}}{\mathbf{h}} * \frac{\mathbf{f(products)}}{\mathbf{f(educts)}} \tag{26}$$

Upon normalization of mass spectral ion intensities, equation (26) becomes:

$$\mathbf{k^{\#}_{mg}} = \frac{\kappa\, k_B\, T_{coll}}{h} * \frac{norm(products)}{norm(educts)} \quad (27)$$

or:

$$\mathbf{k^{\#}_{mg}} = \frac{\kappa\, k_B\, T_{coll}}{h} * \frac{norm(products)}{100\%-norm\ (products)} \quad (28)$$

or:

$$\ln \mathbf{k^{\#}_{mg}} = \ln\left(\frac{\kappa\, k_B\, T_{coll}}{h} * \frac{norm(products)}{100\%-norm\ (products)}\right) \quad (29)$$

Alternatively:

$$\ln \mathbf{k^{\#}_{mg}} = \ln\left(\frac{\kappa\, k_B\, T_{coll}}{h} * \frac{100\%-norm\ (educts)}{norm(educts)}\right) \quad (30)$$

Substituting equation (17) into equation (29) yields:

$$\ln \mathbf{k^{\#}_{mg}} = \ln\left(\frac{\kappa\, k_B\, (T_{amb} + \frac{x\, m\, e}{3N_{at}\, k_B} * \Delta CV)}{h} * \frac{norm(products)}{100\%-norm\ (products)}\right) \quad (31)$$

Alternatively, substituting equation (17) into equation (30) yields:

$$\ln \mathbf{k^{\#}_{mg}} = \ln\left(\frac{\kappa\, k_B\, (T_{amb} + \frac{x\, m\, e}{3N_{at}\, k_B} * \Delta CV)}{h} * \frac{100\%-norm\ (educts)}{norm(educts)}\right) \quad (32)$$

where:

$k^{\#}_{mg}$ apparent rate constant of dissociation reaction of the mean charge state of multiply charged and accelerated protein - ligand complex ions in the gas phase
κ transmission coefficient; is equal to 1 as the products do not re-cross to the transition state
k_B Boltzmann constant, 1.38064852 x 10^{-23} J/K
T_{amb} absolute ambient temperature, 298 K
x overall kinetic energy converted to internal energy, x = 0.24 [15]
m abundance weighted mean charge state of multiply charged and accelerated protein - ligand complex ions
e fundamental charge, 1.602176634 x 10^{-19} C
N_{at} number of atoms in the protein - ligand complex
ΔCV collision cell voltage difference
h Planck's constant, 6.62607015 x 10^{-34} Js
$norm(educts)$ normalized intensity of educts
$norm(products)$ normalized intensity of products

From the Arrhenius equation, the rate constant $\mathbf{k^{\#}_{m0g}}$ and the apparent energy of activation of protein – ligand complex dissociation $\Delta G^{\#}_{mg}$ can be determined.

$$\mathbf{k^{\#}} = \mathbf{A} * \mathbf{e}^{-\frac{\Delta G^{\#}}{RT}} \quad (33)$$

where:

$\mathbf{k^{\#}}$ apparent rate constant of total reaction
$\mathbf{A}$ pre-exponential factor
$\mathbf{\Delta G^{\#}}$ apparent Gibbs energy of activation.
$\mathbf{R}$ gas constant, 8.314 J/mol•K
$\mathbf{T}$ absolute temperature

or:

$$\ln \mathbf{k^{\#}} = \ln \mathbf{A} - \frac{\Delta G^{\#}}{RT} \quad (34)$$

or:

$$\ln \mathbf{k^{\#}_{mg}} = \ln A - \frac{\Delta G^{\#}_{mg}}{R} * \frac{1}{T_{coll}} \quad (35)$$

where:

$\mathbf{k^{\#}_{mg}}$ apparent rate constant of dissociation reaction of the mean charge state of multiply charged and accelerated protein - ligand complex ions in the gas phase

A pre-exponential factor

$\mathbf{\Delta G^{\#}_{mg}}$ apparent Gibbs energy of activation of the abundance weighted mean charge state of multiply charged and accelerated protein - ligand complex ions in the gas phase

R gas constant, 8.314 J / mol • K

$\mathbf{T_{coll}}$ protein-ligand temperature during collision in the collision cell

Plotting $\mathbf{ln k^{\#}_{mg}}$ as a function of $\frac{\mathbf{1}}{\mathbf{T_{coll}}}$ provides the intercept with the y-axis (cf. Figure 3B), which is **ln A** (pre-exponential factor) and the slope of the line, which is $-\frac{\mathbf{\Delta G^{\#}_{mg}}}{\mathbf{R}}$.

Note, at $\mathbf{T_{coll}} = \mathbf{T_{amb}} = 298$ K it can be concluded from equation (16) that:

$$\mathbf{\Delta CV = 0} \quad (36)$$

Hence, at ΔCV = 0 a calculated value for $\mathbf{k^{\#}_{m0g}}$ is obtained i.e. the apparent rate constant of dissociation of "neutral and resting" protein - ligand complexes.

By applying the Eyring-Polanyi equation and from $\mathbf{k^{\#}_{m0g}}$, $\mathbf{K^{\#}_{D\,m0g}}$ can be determined:

$$\mathbf{ln\,k^{\#}_{m0g}} = \frac{\mathbf{\kappa\, k_B\, T_{amb}}}{\mathbf{h}} * \mathbf{K^{\#}_{D\,m0g}} \quad (37)$$

or:

$$\mathbf{K^{\#}_{D\,m0g}} = \frac{\mathbf{h}}{\mathbf{\kappa\, k_B\, T_{amb}}} * \mathbf{ln\,k^{\#}_{m0g}} \quad (38)$$

where:

$\mathbf{K^{\#}_{D\,m0g}}$ apparent gas phase thermodynamic equilibrium dissociation constant of "neutral and resting" protein - ligand complexes

h Planck's constant, $6.62607015 \times 10^{-34}$ J s

κ transmission coefficient; is equal to 1 as the products do not re-cross to the transition state

$\mathbf{k_B}$ Boltzmann constant, $1.38064852 \times 10^{-23}$ J / K

$\mathbf{T_{amb}}$ absolute ambient temperature, 298 K

$\mathbf{k^{\#}_{m0g}}$ apparent rate constant of dissociation reaction of "neutral and resting" protein - ligand complexes

Thus, at ΔCV=0 the value for $\mathbf{K^{\#}_{D\,m0g}}$, is calculated i.e. the apparent gas phase thermodynamic equilibrium dissociation constants of protein - ligand complex dissociation, corrected for external energy contributions; i.e. of "neutral and resting" protein - ligand complexes.

At last, by applying the van´t Hoff equation, $\mathbf{\Delta G^{\#}_{m0g}}$ can be determined:

$$\mathbf{\Delta G^{\#}_{m0g}} = \mathbf{-R\,T_{amb}\;ln\,K^{\#}_{D\,m0g}} \quad (39)$$

where:

$\mathbf{\Delta G^{\#}_{m0g}}$ apparent Gibbs energy of activation of neutral and resting protein - ligand complexes

R gas constant, 8.314 J / mol • K

$\mathbf{T_{amb}}$ absolute ambient temperature, 298 K

$\mathbf{K^{\#}_{D\,m0g}}$ apparent gas phase thermodynamic equilibrium dissociation constants of neutral and resting protein - ligand complexes

Therefore, at $\mathbf{\Delta CV = 0}$ the value for $\mathbf{\Delta G^{\#}_{m0g}}$ is calculated i.e. the apparent Gibbs energy of activation of neutral and resting protein - ligand complexes.

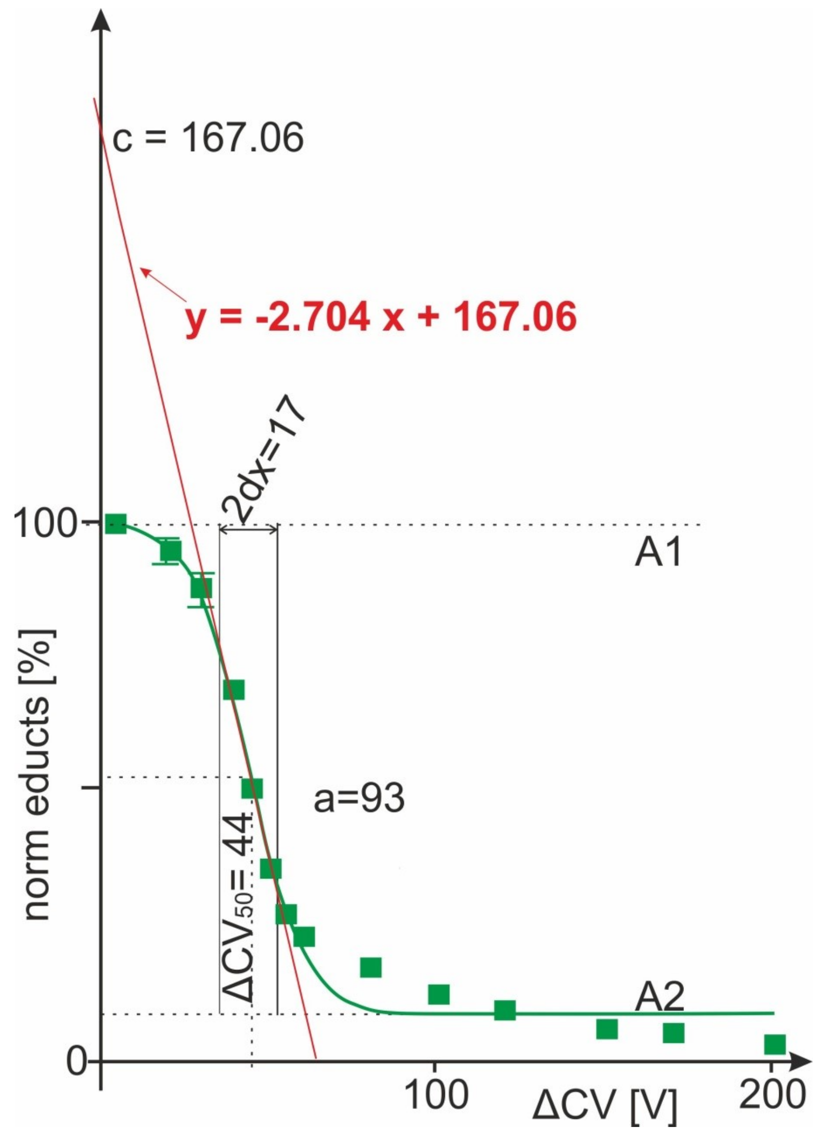

Supplemental figure 1: Course of normalized ion intensities of holo-myoglobin ions ($norm\ (educts)$) as a function of collision cell voltage differences (*ΔCV*). Each data point is the mean of three independent measurements. Vertical bars give standard deviations. The curve was fitted using a Boltzmann function. The tangent line equation is taken from the Boltzmann fit. "a" describes the difference between the highest and lowest data points on the sigmoidal fit. "2dx" is the x-axis interval within which the steepest decline of educt is observed. The center of the 2dx interval is ΔCV_{50}.

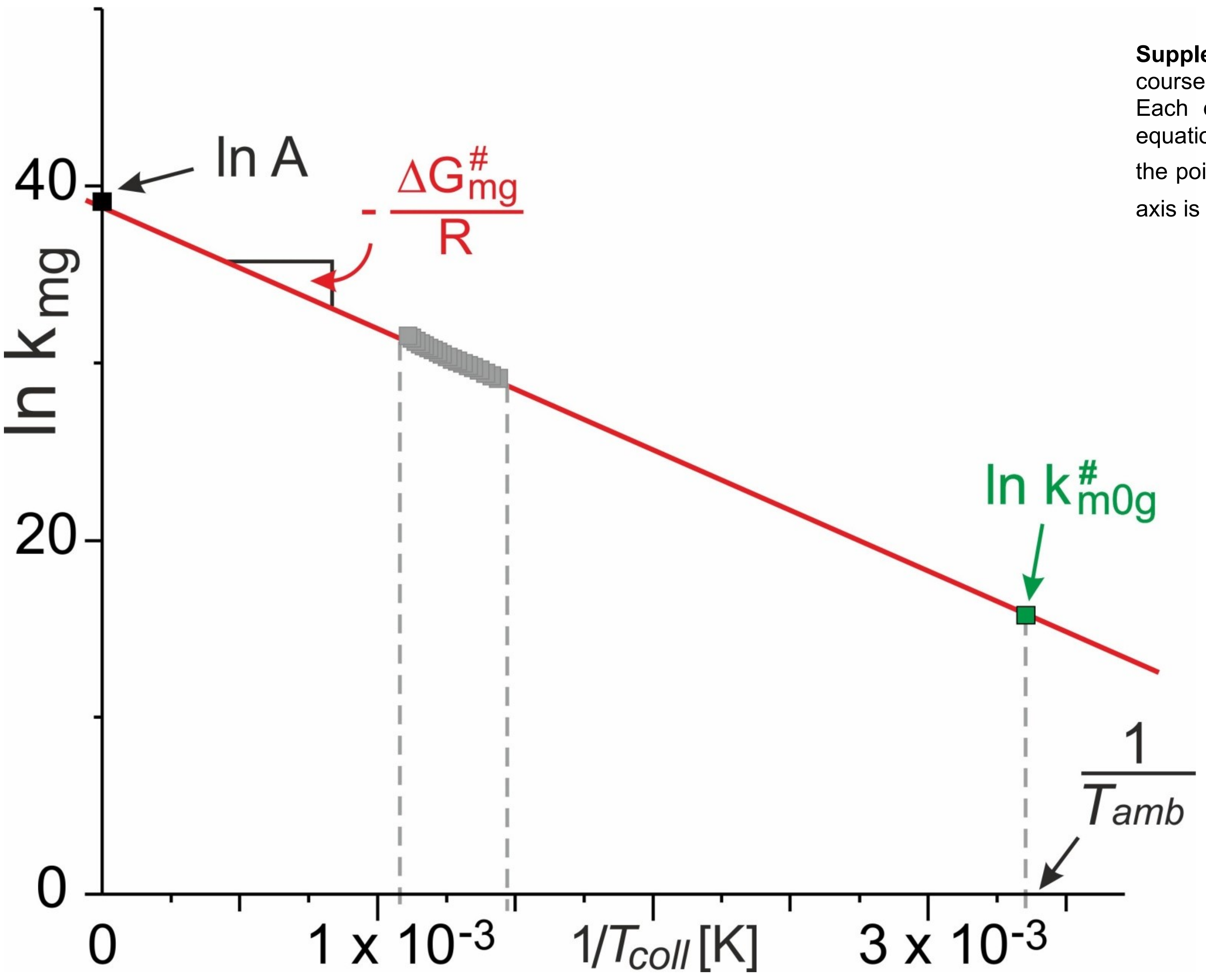

Supplemental figure 2: Arrhenius plot for the course of myoglobin dissociation in the gas phase. Each data point (gray squares) is obtained from equation (32) and the value for $lnk^{\#}_{m0g}$ is taken from the point of the line at $\frac{1}{T_{amb}}$. The intercept with the y-axis is lnA: 39.17.

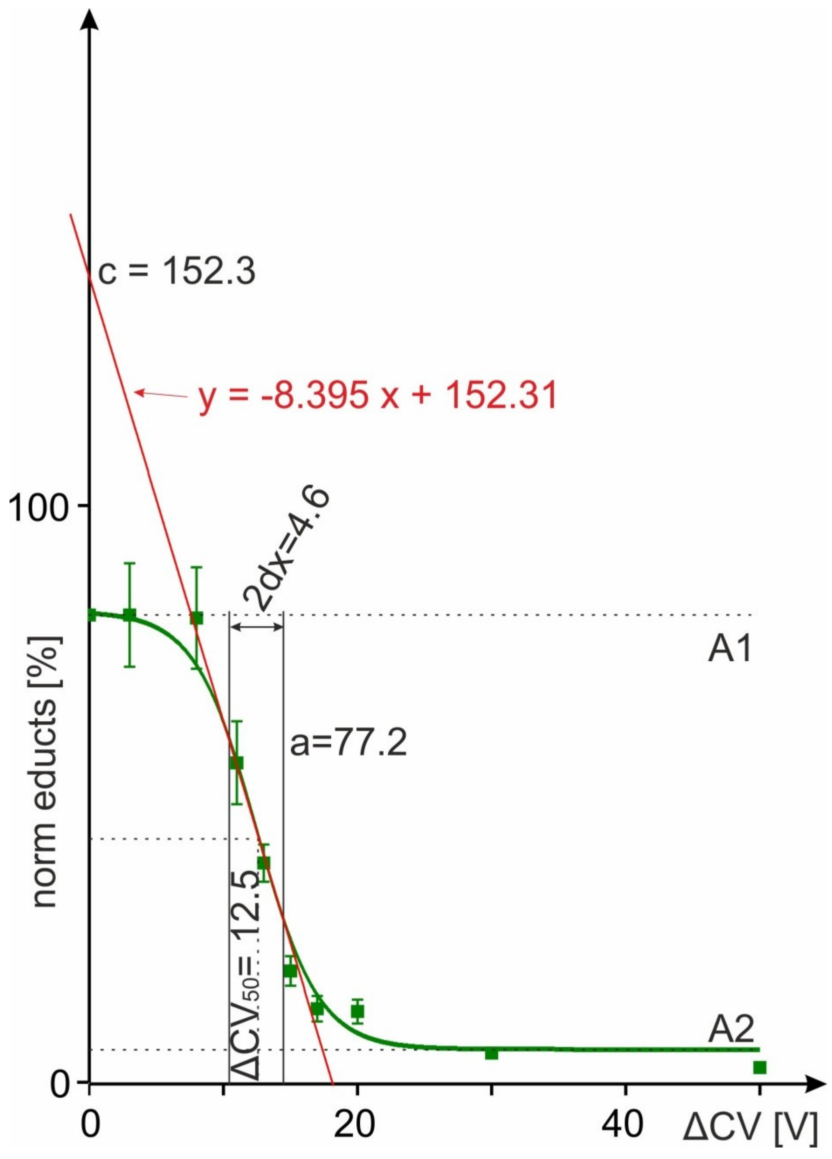

Supplemental figure 3: Course of normalized ion intensities of RNAse S ions ($norm\,(educts)$) as a function of collision cell voltage differences (*ΔCV*). Each data point is the mean of three independent measurements. Vertical bars give standard deviations. The curve was fitted using a Boltzmann function. The tangent line equation is taken from the Boltzmann fit. “a” describes the difference between the highest and lowest data points on the sigmoidal fit. “2dx” is the x-axis interval within which the steepest decline of educt is observed. The center of the 2dx interval is ΔCV_{50}.

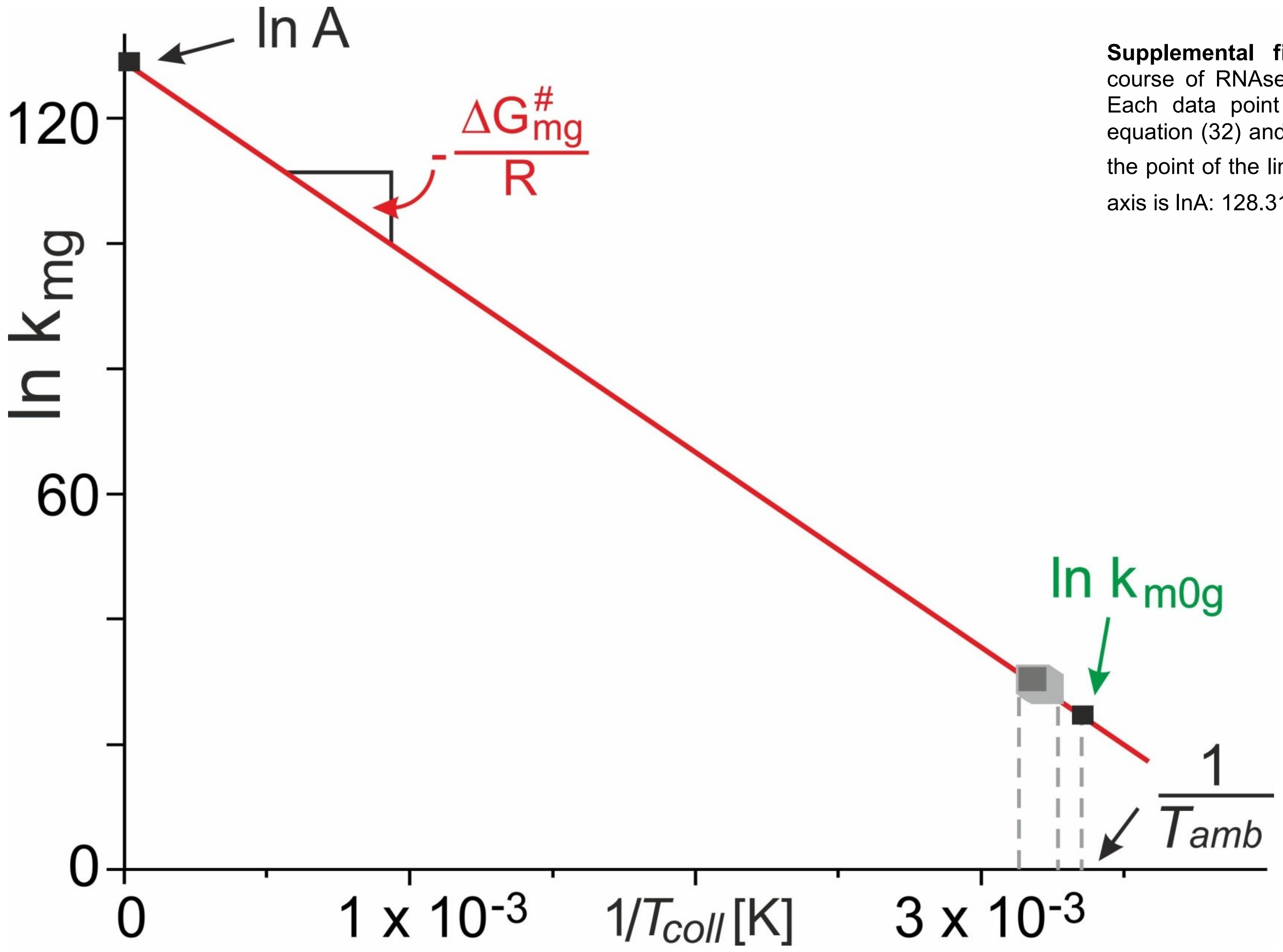

Supplemental figure 4: Arrhenius plot for the course of RNAse S dissociation in the gas phase. Each data point (gray squares) is obtained from equation (32) and the value for $lnk^{\#}_{m0g}$ is taken from the point of the line at $\frac{1}{T_{amb}}$. The intercept with the y-axis is lnA: 128.31.

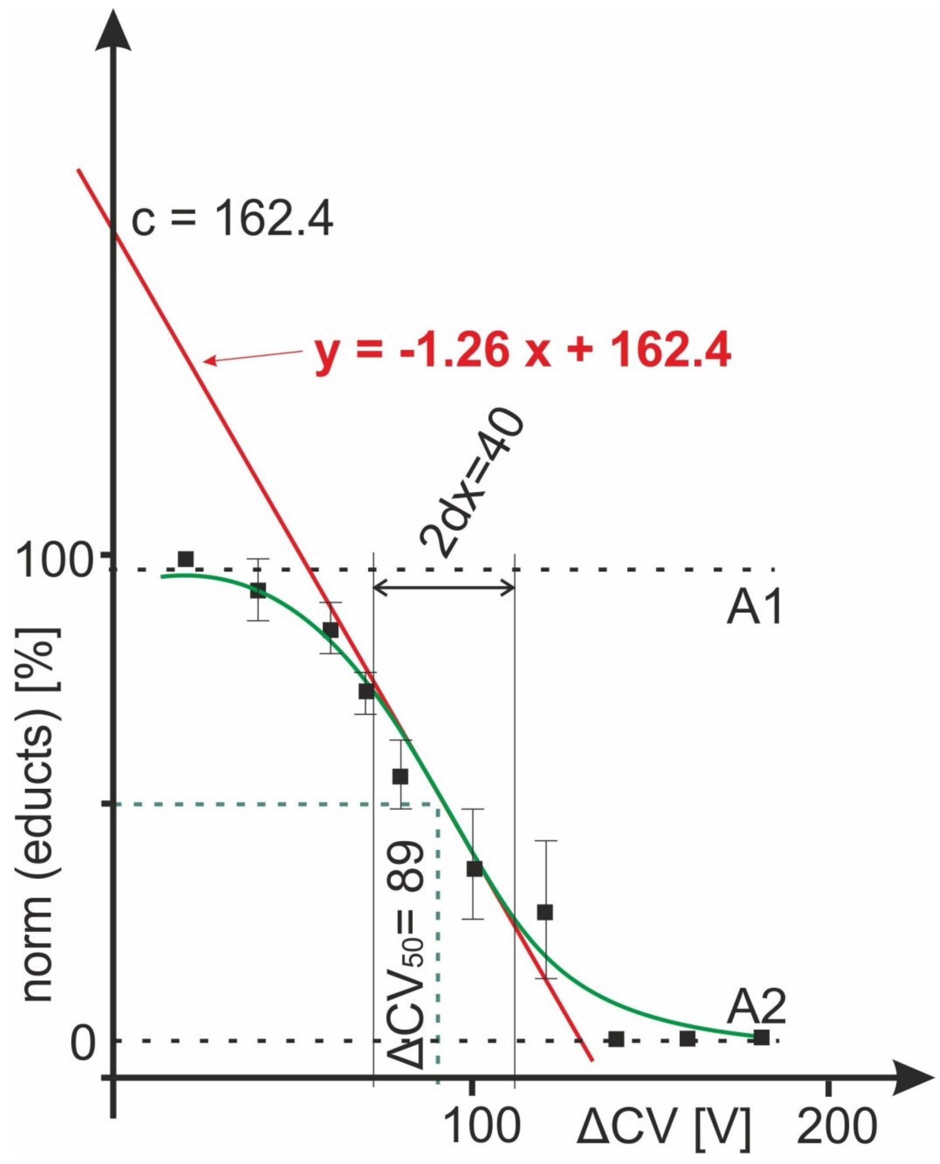

Supplemental figure 5: Course of normalized ion intensities of FLAG-peptide – antiFLAG antibody ions ($norm\,(educts)$) as a function of collision cell voltage differences (*ΔCV*). Each data point is the mean of three independent measurements (example: myoglobin dissociation). Vertical bars give standard deviations. The curve was fitted using a Boltzmann function. The tangent line equation is taken from the Boltzmann fit. “a” describes the difference between the highest and lowest data points on the sigmoidal fit. “dx” is the x-axis interval within which the steepest decline of educt is observed. The center of the 2dx interval is ΔCV_{50}.

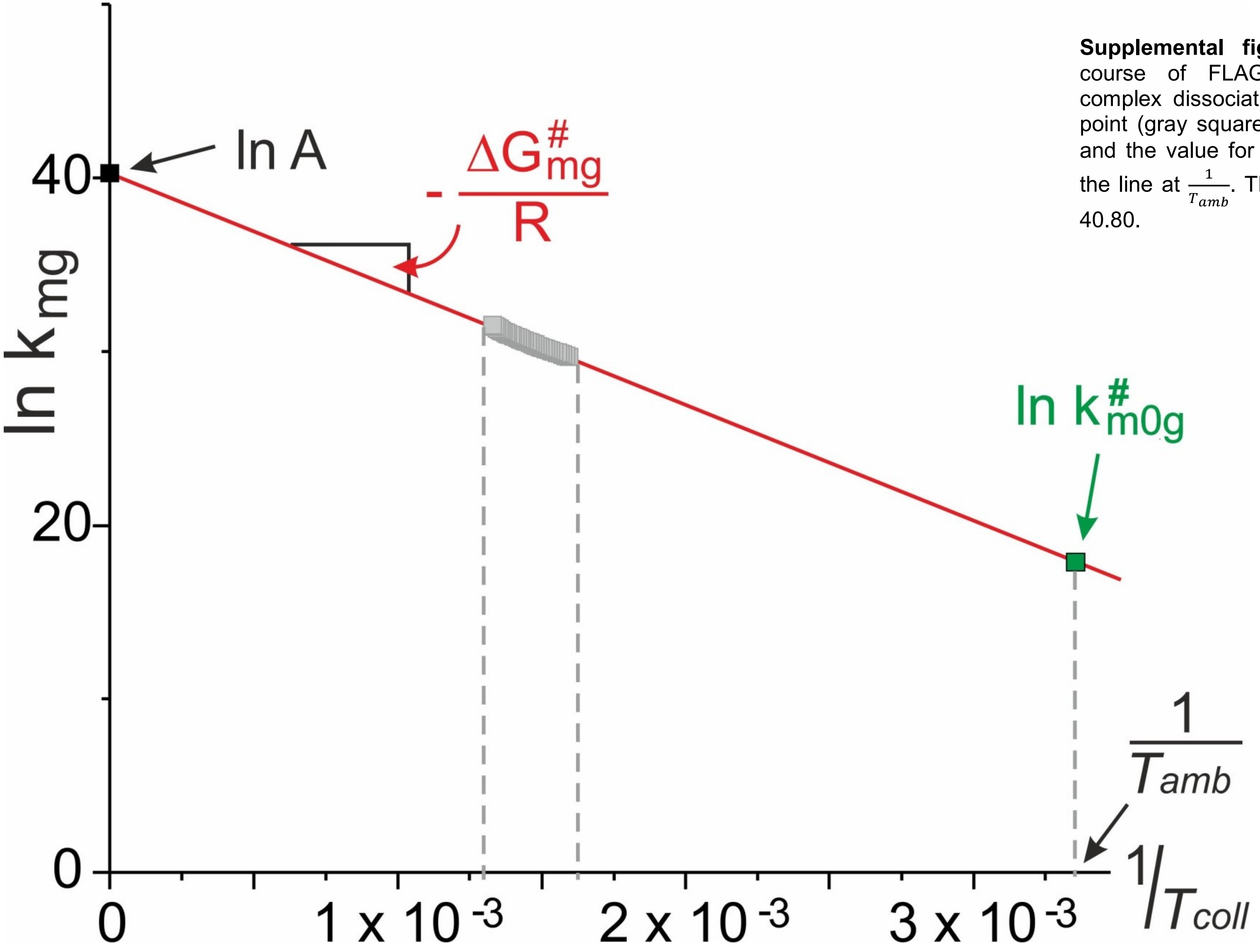

Supplemental figure 6: Arrhenius plot for the course of FLAG-peptide – antiFLAG antibody complex dissociation in the gas phase. Each data point (gray squares) is obtained from equation (32) and the value for $lnk^{\#}_{m0g}$ is taken from the point of the line at $\frac{1}{T_{amb}}$. The intercept with the y-axis is lnA: 40.80.

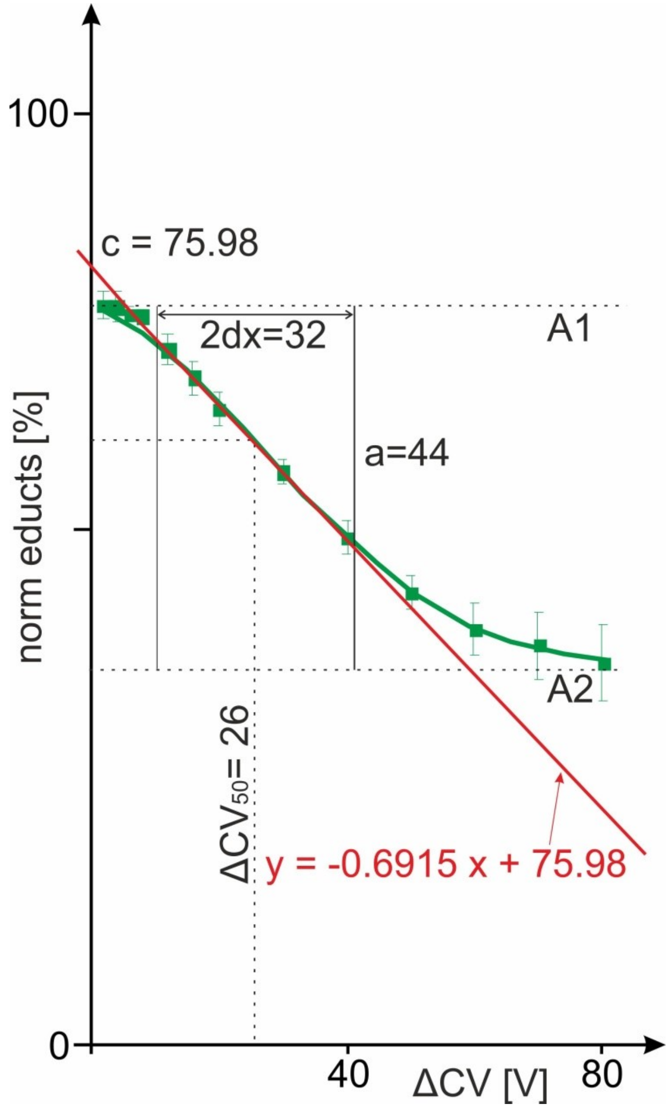

Supplemental figure 7: Course of normalized ion intensities of Troponin I peptide – antiTroponin I antibody complex ($norm\ (educts)$) as a function of collision cell voltage differences (*ΔCV*). Each data point is the mean of two independent measurements. Vertical bars give standard deviations. The curve was fitted using a Boltzmann function. The tangent line equation is taken from the Boltzmann fit. “a” describes the difference between the highest and lowest data points on the sigmoidal fit. “2dx” is the x-axis interval within which the steepest decline of educt is observed. The center of the 2dx interval is ΔCV_{50}.

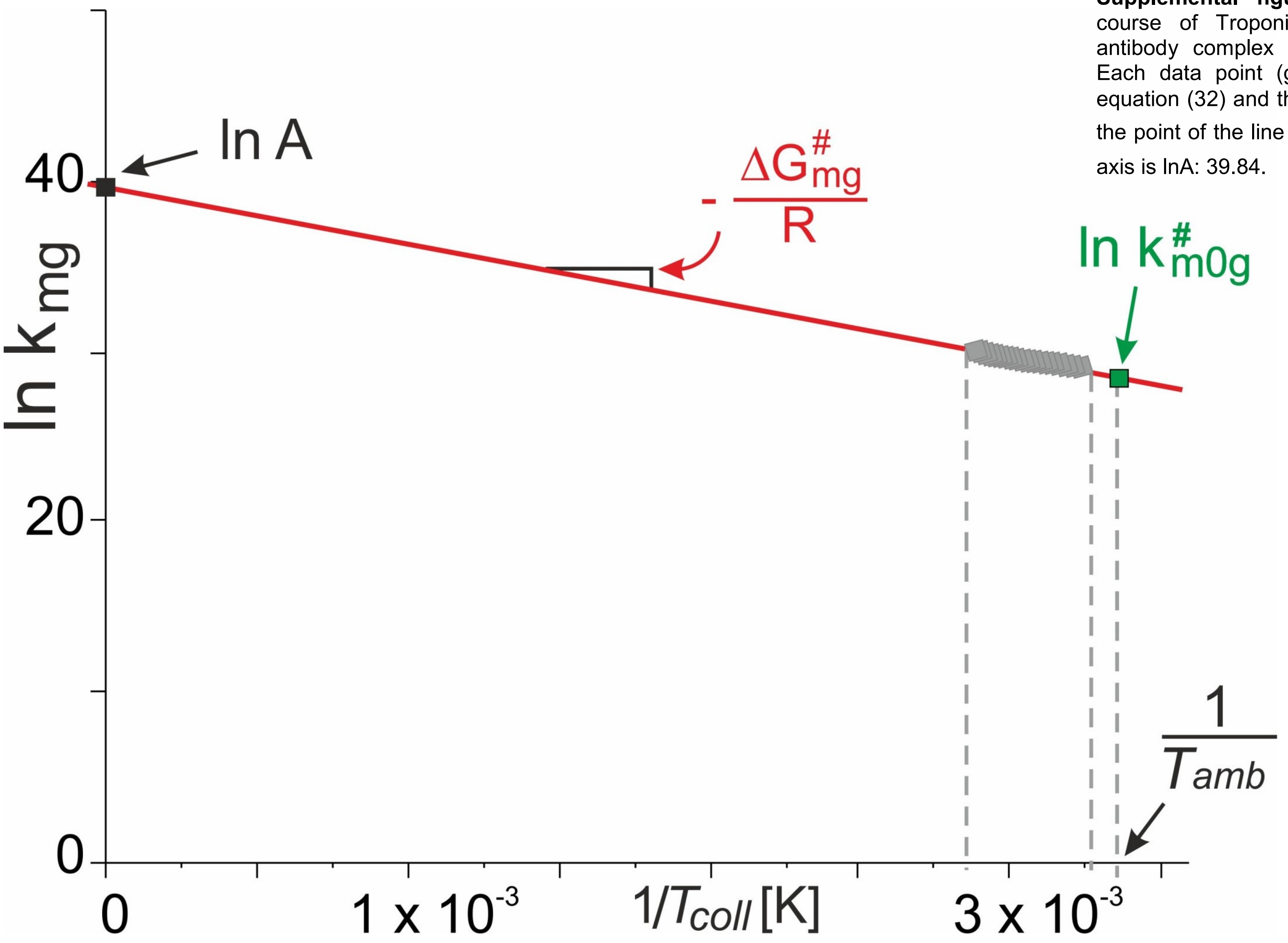

Supplemental figure 8: Arrhenius plot for the course of Troponin I peptide – antiTroponin I antibody complex dissociation in the gas phase. Each data point (gray squares) is obtained from equation (32) and the value for $lnk^{\#}_{m0g}$ is taken from the point of the line at $\frac{1}{T_{amb}}$. The intercept with the y-axis is lnA: 39.84.

Supplemental Table 1: Ion intensities, charge states, and m/z values for myoglobin at various collision cell voltage difference settings.

1st determination

HOLO

ion	m/z	4V	20V	30V	40V	45V	50V	60V	80V	100V	120V	150V	170V	200V
6+	2928	0	0	0	0	0	0	0	0	0	0	0	0	0
7+	2510	28,4	19,2	19,9	18,9	19,6	18,2	14,2	9,21	5,59	5,1	2,8	2,85	1,74
8+	2196	322	166	143	135	142	133	110	85,8	65,5	51	36	27,7	17,6
9+	1952	77,4	34,1	22,5	23,2	27,8	33,5	28,5	22,3	18,4	12,5	9,47	7,62	4,74
10+	1757	0	0	0	0	0	0	0	0	0	0	0	0	0

APO

ion	m/z	4V	20V	30V	40V	45V	50V	60V	80V	100V	120V	150V	170V	200V
5+	3391	0	0	0	0	0	0	0	0	0	0	0	0	0
6+	2826,33	0	0	2,28	2,92	3,89	6,92	15,8	15,6	7,75	4,85	3,28	2,72	1,76
7+	2422,67	0	2,81	4,72	15,2	37	80,6	99,5	78,5	58,2	52,3	40,2	33,6	23,3
8+	2119,93	0	1,06	3,29	11,8	19,4	28	29,8	20,7	21,3	19,7	16,9	12,3	10,1
9+	1884,5	0	0	1	1,84	2,01	2,97	2,11	2,17	2,45	1,77	1,38	1	1

HEME

ion	m/z	4V	20V	30V	40V	45V	50V	60V	80V	100V	120V	150V	170V	200V
1+T	1846,6	0	0	0	0	0	0	0	0	0	0	0	0	0
1+D	1231,4	0	0	0	0	0	0	0	0	0	0	0	0	0
1+	616,2	0	2,99	13	47,4	101	172	241	317	379	393	345	312	262
2+	308,6	0	0	0	0	0	0	0	0	0	0	0	0	0
3+	206,1	0	0	0	0	0	0	0	0	0	0	0	0	0

HEME-Ac

ion	m/z	4V	20V	30V	40V	45V	50V	60V	80V	100V	120V	150V	170V	200V
1+T	1669,6	0	0	0	0	0	0	0	0	0	0	0	0	0
1+D	1113,4	0	0	0	0	0	0	0	0	0	0	0	0	0
1+	557,2	0	0	0	0	0	0	0	2,69	21,6	67,6	140	168	182
2+	279,1	0	0	0	0	0	0	0	0	0	0	0	0	0
3+	186,4	0	0	0	0	0	0	0	0	0	0	0	0	0

HEME-2Ac

ion	m/z	4V	20V	30V	40V	45V	50V	60V	80V	100V	120V	150V	170V	200V
1+T	1492,6	0	0	0	0	0	0	0	0	0	0	0	0	0
1+D	995,4	0	0	0	0	0	0	0	0	0	0	0	0	0
1+	498,2	0	0	0	0	0	0	0	0	0	0	47,6	90	156
2+	249,6	0	0	0	0	0	0	0	0	0	0	0	0	0
3+	166,7	0	0	0	0	0	0	0	0	0	0	0	0	0

(T=trimer; D=dimer)

Supplemental Table 1: continued

2nd determination

HOLO

**ion**	**m/z**	**4V**	**20V**	**30V**	**40V**	**45V**	**50V**	**55V**	**60V**	**80V**	**100V**	**120V**	**150V**	**170V**	**200V**
**6+**	2928	0	0	0	0	0	0	0	0	0	0	0	0	0	0
**7+**	2510	29	23,5	17,5	22,8	24,4	22,8	15,3	17,4	13	9,16	6,47	4,81	3,81	2,11
**8+**	2196	289	164	136	176	172	170	151	143	122	91,6	73,3	52,8	38,3	25,7
**9+**	1952	62	26,5	23,8	33,8	35,5	39	34,1	38,3	31,8	25	20	12,2	10,5	6,92
**10+**	1757	0	0	0	0	0	0	0	0	0	0	0	0	0	0

APO

**ion**	**m/z**	**4V**	**20V**	**30V**	**40V**	**45V**	**50V**	**55V**	**60V**	**80V**	**100V**	**120V**	**150V**	**170V**	**200V**
**5+**	3391	0	0	0	0	0	0	0	0	0	0	0	0	0	0
**6+**	2826,33	0	0	2,97	4,01	5,42	9,39	15,3	20,4	21,4	10,7	6,5	4,95	3,48	2,8
**7+**	2422,67	0	4,92	7,45	18,9	46,3	87	123	146	103	79,2	68,4	56	49	33,2
**8+**	2119,93	0	1,67	5,94	18,8	27,3	36,2	43,7	47,8	29,3	29,6	30,1	25,5	22,4	15,7
**9+**	1884,5	0	0	1,17	2,35	4,03	3,31	4,01	3,15	3,22	3,02	3,05	2,02	1,27	1,35

HEME

**ion**	**m/z**	**4V**	**20V**	**30V**	**40V**	**45V**	**50V**	**55V**	**60V**	**80V**	**100V**	**120V**	**150V**	**170V**	**200V**
**1+T**	1846,6	0	0	0	0	0	0	0	0	0	0	0	0	0	0
**1+D**	1231,4	0	0	0	0	0	0	0	0	0	0	0	0	0	0
**1+**	616,2	0	6,16	17,8	58,8	127	211	290	353	488	558	560	518	466	366
**2+**	308,6	0	0	0	0	0	0	0	0	0	0	0	0	0	0
**3+**	206,1	0	0	0	0	0	0	0	0	0	0	0	0	0	0

HEME-Ac

**ion**	**m/z**	**4V**	**20V**	**30V**	**40V**	**45V**	**50V**	**55V**	**60V**	**80V**	**100V**	**120V**	**150V**	**170V**	**200V**
**1+T**	1669,6	0	0	0	0	0	0	0	0	0	0	0	0	0	0
**1+D**	1113,4	0	0	0	0	0	0	0	0	0	0	0	0	0	0
**1+**	557,2	0	0	0	0	0	0	0	0	4,29	45	104	232	264	335
**2+**	279,1	0	0	0	0	0	0	0	0	0	0	0	0	0	0
**3+**	186,4	0	0	0	0	0	0	0	0	0	0	0	0	0	0

HEME-2Ac

**ion**	**m/z**	**4V**	**20V**	**30V**	**40V**	**45V**	**50V**	**55V**	**60V**	**80V**	**100V**	**120V**	**150V**	**170V**	**200V**
**1+T**	1492,6	0	0	0	0	0	0	0	0	0	0	0	0	0	0
**1+D**	995,4	0	0	0	0	0	0	0	0	0	0	0	0	0	0
**1+**	498,2	0	0	0	0	0	0	0	0	0	0	0	89,5	160	311
**2+**	249,6	0	0	0	0	0	0	0	0	0	0	0	0	0	0
**3+**	166,7	0	0	0	0	0	0	0	0	0	0	0	0	0	0

(T=trimer; D=dimer)

Supplemental Table 1: continued

3rd determination

HOLO

**ion**	**m/z**	**4V**	**20V**	**30V**	**40V**	**45V**	**50V**	**55V**	**60V**	**80V**	**100V**	**120V**	**150V**	**170V**	**200V**
**6+**	2928	0	0	0	0	0	0	0	0	0	0	0	0	0	0
**7+**	2510	24,7	19,8	21,7	22,2	20,8	18,5	14,2	11,7	9,06	5,71	5,02	3,92	6,68	4,28
**8+**	2196	255	141	155	159	150	131	110	103	81,1	56,4	45,9	33,6	27,7	17,2
**9+**	1952	55,9	25,1	32,7	30,2	31,1	28,1	26,6	22,6	20,4	14,4	12,1	9,37	27,7	17,2
**10+**	1757	0	0	0	0	0	0	0	0	0	0	0	0	0	0

APO

**ion**	**m/z**	**4V**	**20V**	**30V**	**40V**	**45V**	**50V**	**55V**	**60V**	**80V**	**100V**	**120V**	**150V**	**170V**	**200V**
**5+**	3391	0	0	0	0	0	0	0	0	0	0	0	0	0	0
**6+**	2826,33	0	0	2,46	3,57	4,68	8,72	11,4	15,8	15,7	7,01	4,41	4,32	2,34	2,05
**7+**	2422,67	0	3,48	4,58	18,1	41	71	88,7	98,6	72,8	54,9	46,6	40	28,1	23,3
**8+**	2119,93	0	1,42	3,89	12,3	18,3	24	23,1	28,6	21,7	19,2	20	15,1	12,6	9,85
**9+**	1884,5	0	0	1	2,09	1,58	2,15	1,8	2,34	2,62	1,65	2,01	1,52	1,31	1

HEME

**ion**	**m/z**	**4V**	**20V**	**30V**	**40V**	**45V**	**50V**	**55V**	**60V**	**80V**	**100V**	**120V**	**150V**	**170V**	**200V**
**1+T**	1846,6	0	0	0	0	0	0	0	0	0	0	0	0	0	0
**1+D**	1231,4	0	0	0	0	0	0	0	0	0	0	0	0	0	0
**1+**	616,2	0	5,3	12,3	50,1	103	165	209	249	319	364	384	358	297	252
**2+**	308,6	0	0	0	0	0	0	0	0	0	0	0	0	0	0
**3+**	206,1	0	0	0	0	0	0	0	0	0	0	0	0	0	0

HEME-Ac

**ion**	**m/z**	**4V**	**20V**	**30V**	**40V**	**45V**	**50V**	**55V**	**60V**	**80V**	**100V**	**120V**	**150V**	**170V**	**200V**
**1+T**	1669,6	0	0	0	0	0	0	0	0	0	0	0	0	0	0
**1+D**	1113,4	0	0	0	0	0	0	0	0	0	0	0	0	0	0
**1+**	557,2	0	0	0	0	0	0	0	0	2,73	19,7	75,9	143	173	190
**2+**	279,1	0	0	0	0	0	0	0	0	0	0	0	0	0	0
**3+**	186,4	0	0	0	0	0	0	0	0	0	0	0	0	0	0

HEME-2Ac

**ion**	**m/z**	**4V**	**20V**	**30V**	**40V**	**45V**	**50V**	**55V**	**60V**	**80V**	**100V**	**120V**	**150V**	**170V**	**200V**
**1+T**	1492,6	0	0	0	0	0	0	0	0	0	0	0	0	0	0
**1+D**	995,4	0	0	0	0	0	0	0	0	0	0	0	0	0	0
**1+**	498,2	0	0	0	0	0	0	0	0	0	0	0	42,9	97,8	160
**2+**	249,6	0	0	0	0	0	0	0	0	0	0	0	0	0	0
**3+**	166,7	0	0	0	0	0	0	0	0	0	0	0	0	0	0

(T=trimer; D=dimer)

Supplemental Table 2: Apex heights and mean charge states of educt and product ion signals upon gas phase dissociation of myoglobin.

**ΔCV [V]**	**1st determination a)**						**2nd determination a)**						**3rd determination a)**					
	m+	h	n+	i	p+	j	m+	h	n+	i	p+	j	m+	h	n+	i	p+	j
**4**	8,1	322	1	0	-	0	8,1	289,4	1	0	-	0	8,1	255,2	1	0	-	0
**20**	8,1	166,7	1	3	7,3	3,3	8	167,8	1	6,2	7,3	5,4	8	143,3	1	5,4	7,3	3,8,0
**30**	8	146,4	1	13,2	7,3	4,8	8	137,9	1	18,1	7,3	7,8	8,1	156,3	1	12,5	7,3	4,8
**40**	8	137,5	1	48,1	7,4	16,5	8	177,7	1	59,7	7,4	18,7	8	161,1	1	50,9	7,4	18,9
**45**	8	143,6	1	102,6	7,3	38,2	8	173,7	1	129	7,4	48,4	8,1	151,3	1	104,6	7,3	41,6
**50**	8,1	133,3	1	174,7	7,2	81,2	8,1	170,7	1	214,3	7,2	87,9	8,1	132	1	167,5	7,2	71
**55**	n.d.	n.d.	n.d.	n.d.	n.d.	n.d.	8,1	151,1	1	294,5	7,2	123,2	8,1	110,3	1	212,2	7	88,9
**60**	8,1	110,1	1	244,6	7,1	99,7	8,1	143,1	1	358,4	7,2	146	8,1	103,3	1	252,8	7,1	98,9
**80**	8,1	85,8	1	324,6	7,1	79,5	8,1	122	1	499,9	7,1	104,1	8,1	81,1	1	326,7	7,1	73,5
**100**	8,1	65,7	1	406,5	7,2	58,3	8,1	91,7	1	611,8	7,2	79,3	8,1	56,4	1	389,6	7,2	54,9
**120**	8,1	51	1	466,9	7,2	52,8	8,1	73,4	1	674,2	7,3	69,7	8,1	45,9	1	467	7,3	47,4
**150**	8,1	36,1	1	539	7,3	41	8,1	52,8	1	852,5	7,3	57,4	8,1	33,6	1	552,3	7,2	40,2
**170**	8,1	27,7	1	576,7	7,2	34	8,1	38,3	1	903,8	7,3	50,7	8,3	27,7	1	576,6	7,3	28,8
**200**	8,1	17,6	1	607	7,3	23,9	8,1	25,7	1	1028	7,3	34,2	8,3	17,2	1	611,3	7,3	23,7

a) arbitrary units; cf. Figure 4; n.d.: not determined

Supplemental Table 3: Ion intensities, charge states, and m/z values for RNAse S at various collision cell voltage difference settings.

1st determination

RNAse S Complex 1-19...21-124

ion	m/z	3V	8V	11V	13V	15V	17V	20V	30V	50V
5+	2727,90	1,83	1,01	2,84	3,63	4,82	3,14	3,89	3,44	1,46
6+	2272,97	51,10	36,30	67,40	109,00	124,00	99,20	98,20	49,80	19,10
7+	1948,36	150,00	116,00	166,00	135,00	98,60	46,30	32,10	20,50	9,56
8+	1704,93	0,90	0,58	0,67	0,00	0,29	0,00	0,28	0,16	0,28
9+	1515,49	0,00	0,00	0,00	0,00	0,00	0,00	0,00	0,00	0,12

RNAse S Complex 1-20...22-124

ion	m/z	3V	8V	11V	13V	15V	17V	20V	30V	50V
5+	2709,68	0,00	0,00	0,00	0,93	0,80	1,41	2,31	1,84	0,86
6+	2258,30	31,20	20,80	0,00	67,30	71,80	55,60	57,00	22,90	4,22
7+	1935,95	78,10	64,70	48,30	70,50	49,90	23,20	20,30	12,70	5,69
8+	1693,60	0,56	0,46	0,18	0,00	0,00	0,11	0,30	0,00	0,00
9+	1505,78	0,00	0,00	0,16	0,00	0,00	0,00	0,00	0,00	0,00

S Protein 21-124

ion	m/z	3V	8V	11V	13V	15V	17V	20V	30V	50V
3+	3845,75	0,00	0,00	0,00	0,00	0,00	0,00	0,00	0,00	0,00
4+	2884,76	0,00	0,00	0,06	0,00	0,00	0,36	1,24	42,80	44,80
5+	2308,44	0,00	5,77	53,80	178,00	294,00	270,00	303,00	355,00	392,00
6+	1923,71	6,00	5,26	8,56	7,47	8,35	5,36	6,54	6,41	11,30
7+	1648,71	0,00	0,00	1,03	0,00	0,20	0,13	0,13	0,21	0,16

S Protein 22-124

ion	m/z	3V	8V	11V	13V	15V	17V	20V	30V	50V
3+	3816,46	0,00	0,00	0,00	0,00	0,00	0,00	0,00	0,00	0,00
4+	2862,45	0,00	0,00	0,00	0,00	0,00	0,40	1,10	29,20	28,40
5+	2289,00	0,00	12,80	42,80	120,00	190,00	175,00	194,00	217,00	241,00
6+	1908,50	1,06	1,00	2,43	3,15	4,09	3,04	2,62	1,01	5,71
7+	1636,20	0,00	0,00	0,00	0,00	0,00	0,00	0,17	0,06	0,16

S Peptide 1-19

ion	m/z	3V	8V	11V	13V	15V	17V	20V	30V	50V
1+D	4189,30	0,00	0,00	0,00	0,00	0,00	0,00	0,00	0,00	0,00
1+	2095,35	0,00	0,00	0,00	0,00	0,00	0,00	0,00	2,52	5,61
2+	1048,18	0,18	1,53	39,30	153,00	258,00	261,00	257,00	377,00	430,00
3+	699,15	0,00	1,00	0,00	0,09	0,34	0,21	0,33	0,68	0,25
4+	524,53	0,00	0,00	0,00	0,00	0,00	0,00	0,00	0,00	0,00

S Peptide 1-20

ion	m/z	3V	8V	11V	13V	15V	17V	20V	30V	50V
1+D	4330,36	0,00	0,00	0,00	0,00	0,00	0,00	0,00	0,00	0,00
1+	2166,18	0,00	0,00	0,00	0,00	0,00	0,00	0,00	1,19	1,05
2+	1083,57	**0,06**	0,34	9,50	42,40	78,90	83,00	95,90	141,00	157,00
3+	722,89	0,00	0,00	0,00	0,00	0,22	0,19	0,07	0,00	0,06
4+	542,30	0,00	0,00	0,00	0,00	0,00	0,00	0,00	0,00	0,00

D=Dimer

Supplemental Table 3: continued

2nd determination

RNAse S Complex 1-19...21-124

ion	m/z	3V	8V	11V	13V	15V	17V	20V	30V	50V
5+	2727,2	0,46	0,70	1,06	0,75	0,94	1,44	2,11	1,31	1,12
6+	2272,7	108,00	134,00	153,00	114,00	122,00	115,00	177,00	54,10	22,00
7+	1948,2	177,00	213,00	161,00	71,30	41,90	28,20	35,80	13,30	7,51
8+	1705	1,29	1,05	0,57	0,59	0,42	0,00	0,25	0,09	0,17
9+	1515,5	0,00	0,00	0,00	0,00	0,00	0,00	0,00	0,00	0,00

RNAse S Complex 1-20...22-124

ion	m/z	3V	8V	11V	13V	15V	17V	20V	30V	50V
5+	2709,6	0,26	0,36	0,87	0,46	1,22	1,40	1,51	1,50	0,70
6+	2258	57,80	75,20	87,50	64,80	68,30	64,00	97,50	26,40	8,64
7+	1935,7	96,20	111,00	86,70	39,70	21,90	15,70	20,40	7,22	4,65
8+	1693,9	0,68	0,68	0,50	0,13	0,35	0,19	0,27	0,22	0,20
9+	1505,8	0,00	0,00	0,00	0,00	0,00	0,00	0,00	0,00	0,15

S Protein 21-124

ion	m/z	3V	8V	11V	13V	15V	17V	20V	30V	50V
3+	3845,8	0,00	0,00	0,00	0,00	0,00	0,00	0,00	0,00	0,00
4+	2884,7	0,00	0,00	0,00	0,00	0,00	1,01	2,20	39,40	46,00
5+	2308,6	24,80	32,00	78,90	121,00	151,00	151,00	250,00	188,00	185,00
6+	1923,3	8,19	9,08	8,22	4,29	3,32	2,57	3,49	4,23	3,74
7+	1648,8	0,00	0,00	0,00	0,00	0,23	0,00	0,00	0,00	0,22

S Protein 22-124

ion	m/z	3V	8V	11V	13V	15V	17V	20V	30V	50V
3+	3816,46	0,00	0,00	0,00	0,00	0,00	0,00	0,00	0,00	0,00
4+	2862,59	0,00	0,25	0,00	0,00	0,00	0,71	2,11	24,60	27,90
5+	2288,33	51,00	64,20	76,60	95,80	104,00	108,00	171,00	125,00	115,00
6+	1908,23	1,70	2,37	1,93	1,26	1,30	1,36	1,53	2,23	2,57
7+	1636,20	0,00	0,18	0,00	0,00	0,00	0,32	0,00	0,00	0,18

S Peptide 1-19

ion	m/z	3V	8V	11V	13V	15V	17V	20V	30V	50V
1+D	4189,30	0,00	0,00	0,00	0,00	0,00	0,00	0,00	0,00	0,00
1+	2095,15	0,00	0,00	0,00	0,00	0,00	0,00	0,00	4,19	7,46
2+	1048,59	**0,29**	7,58	80,00	142,00	189,00	195,00	281,00	379,00	405,00
3+	699,05	0,06	0,00	0,00	0,00	0,11	0,17	0,19	0,19	0,00
4+	524,53	0,00	0,00	0,00	0,00	0,00	0,00	0,00	0,00	0,00

S Peptide 1-20

ion	m/z	3V	8V	11V	13V	15V	17V	20V	30V	50V
1+D	4330,36	0,00	0,00	0,00	0,00	0,00	0,00	0,00	0,00	0,00
1+	2166,18	0,00	0,00	0,00	0,00	0,00	0,00	0,00	0,00	2,28
2+	1083,59	0,18	1,67	21,40	39,40	64,90	71,40	107,00	145,00	157,00
3+	722,72	0,00	0,00	0,12	0,00	0,00	0,17	0,00	0,00	0,00
4+	542,30	0,00	0,00	0,00	0,00	0,00	0,00	0,00	0,00	0,00

D=Dimer

Supplemental Table 3: continued

3rd determination

RNAse S Complex 1-19...21-124

ion	m/z	3V	8V	11V	13V	15V	17V	20V	30V	50V
5+	2727,6	1,59	1,57	2,88	1,07	3,15	2,84	3,68	1,96	0,59
6+	2272,9	130,00	183,00	234,00	195,00	229,00	178,00	269,00	52,70	14,60
7+	1948,4	215,00	281,00	278,00	127,00	84,00	50,40	60,30	15,70	5,57
8+	1705,2	1,03	0,39	0,31	0,30	0,33	0,38	0,75	0,37	0,29
9+	1515,5	0,00	0,00	0,06	0,00	0,13	0,15	0,00	0,12	0,18

RNAse S Complex 1-20...22-124

ion	m/z	3V	8V	11V	13V	15V	17V	20V	30V	50V
5+	2709,68	0,62	0,58	0,65	1,56	1,54	1,70	2,75	1,64	0,34
6+	2258,39	79,60	109,00	137,00	116,00	135,00	108,00	153,00	25,80	6,34
7+	1935,92	119,00	161,00	155,00	69,9	44,50	27,10	33,30	8,68	4,14
8+	1694,24	0,77	0,69	0,28	0,38	0,28	0,13	0,39	0,41	0,26
9+	1505,76	0,00	0,00	0,00	0,00	0,00	0,07	0,00	0,11	0,18

S Protein 21-124

ion	m/z	3V	8V	11V	13V	15V	17V	20V	30V	50V
3+	3845,75	0,00	0,00	0,00	0,00	0,00	0,00	0,00	0,00	0,00
4+	2884,90	0,22	0,12	0,00	0,38	0,71	0,94	3,58	40,50	33,60
5+	2308,05	10,40	15,50	92,40	184,00	288,00	259,00	429,00	228,00	156,00
6+	1923,58	8,76	12,60	11,20	6,18	5,21	4,20	6,46	3,63	3,94
7+	1648,87	0,22	0,00	0,00	0,24	0,12	0,24	0,57	0,33	0,12

S Protein 22-124

ion	m/z	3V	8V	11V	13V	15V	17V	20V	30V	50V
3+	3816,46	0,00	0,00	0,00	0,00	0,00	0,00	0,00	0,00	0,00
4+	2862,33	0,21	0,30	0,00	0,35	0,73	0,87	2,65	26,80	22,50
5+	2288,35	34,90	39,80	75,80	125,00	189,00	174,00	278,00	140,00	98,90
6+	1908,24	1,03	1,67	1,72	1,21	2,01	1,84	2,82	2,38	1,88
7+	1636,20	0,00	0,06	0,00	0,18	0,13	0,00	0,21	0,20	0,00

S Peptide 1-19

ion	m/z	3V	8V	11V	13V	15V	17V	20V	30V	50V
1+D	4189,30	0,00	0,00	0,00	0,00	0,00	0,00	0,00	0,00	0,00
1+	2095,15	0,00	0,00	0,00	0,00	0,00	0,00	4,36	3,58	5,92
2+	1048,07	0,21	6,41	71,10	160,00	253,00	270,00	493,00	372,00	362,00
3+	699,05	0,00	0,00	0,06	0,00	0,00	0,31	0,48	0,00	0,00
4+	524,53	0,00	0,00	0,00	0,00	0,00	0,00	0,00	0,00	0,00

S Peptide 1-20

ion	m/z	3V	8V	11V	13V	15V	17V	20V	30V	50V
1+D	4330,36	0,00	0,00	0,00	0,00	0,00	0,00	0,00	0,00	0,00
1+	2166,18	0,00	0,00	0,00	0,00	0,00	0,00	0,00	0,00	2,14
2+	1083,60	0,00	1,04	18,40	47,20	90,50	100,00	190,00	144,00	141,00
3+	722,72	0,00	0,00	0,00	0,00	0,00	0,00	0,24	0,00	0,23
4+	542,30	0,00	0,00	0,00	0,00	0,00	0,00	0,00	0,00	0,00

D=Dimer

Supplemental Table 4: Apex heights and mean charge states of educt and product ion signals upon gas phase dissociation of RNAse S.

1st determination [a)]

ΔCV [V]	RNAse S 1-19..21-124		RNAse S 1-20..22-124		S-Protein 21-124		S-Protein 22-124		S-Peptide 1-19		S-Peptide 1-20	
	$m_{(1)}+$	$h_{(1)}$	$m_{(2)}+$	$h_{(2)}$	$p_{(1)}+$	$j_{(1)}$	$p_{(2)}+$	$j_{(2)}$	$n_{(1)}+$	$i_{(1)}$	$n_{(2)}+$	$i_{(2)}$
**3**	6,7	150,63	6,7	79,04	6,0	6,00	6,0	1,06	2,0	0,18	2,0	0,07
**8**	6,7	117,42	6,8	65,32	5,5	5,92	5,1	12,84	2,4	1,51	2,0	0,35
**11**	6,7	169,46	7,0	48,58	5,2	53,28	5,1	42,85	2,0	40,45	2,0	9,78
**13**	6,5	141,61	6,5	73,14	5,0	179,10	5,0	120,54	2,0	157,44	2,0	43,64
**15**	6,5	125,54	6,4	73,50	5,0	295,43	5,0	190,09	2,0	265,43	2,0	81,14
**17**	6,3	101,91	6,3	56,47	5,0	271,48	5,0	176,16	2,0	268,56	2,0	85,37
**20**	6,2	98,79	6,2	56,98	5,0	305,32	5,0	195,58	2,0	264,41	2,0	98,68
**30**	6,2	51,54	6,3	23,88	4,9	358,12	4,9	224,21	2,0	386,44	2,0	144,48
**50**	6,3	19,91	6,5	5,74	4,9	397,52	4,9	244,36	2,0	439,48	2,0	161,00

[a)] arbitrary units; cf. Figure 5

2nd determination [a)]

ΔCV [V]	RNAse S 1-19..21-124		RNAse S 1-20..22-124		S-Protein 21-124		S-Protein 22-124		S-Peptide 1-19		S-Peptide 1-20	
	$m_{(1)}+$	$h_{(1)}$	$m_{(2)}+$	$h_{(2)}$	$p_{(1)}+$	$j_{(1)}$	$p_{(2)}+$	$j_{(2)}$	$n_{(1)}+$	$i_{(1)}$	$n_{(2)}+$	$i_{(2)}$
**3**	6,6	178,50	6,6	96,00	5,3	96,00	5,0	51,30	2,1	0,30	2,0	0,18
**8**	6,6	217,22	6,6	111,64	5,2	111,64	5,0	64,62	2,0	7,81	2,0	1,72
**11**	6,5	165,55	6,5	89,17	5,1	89,17	5,0	78,49	2,0	82,33	2,0	21,99
**13**	6,4	116,12	6,4	65,55	5,0	65,55	5,0	96,26	2,0	146,14	2,0	96,26
**15**	6,3	123,01	6,2	69,31	5,0	69,31	5,0	104,49	2,0	194,47	2,0	66,80
**17**	6,2	119,16	6,2	64,79	5,0	64,79	5,0	108,77	2,0	200,63	2,0	73,43
**20**	6,2	177,28	6,2	99,01	5,0	99,01	5,0	173,30	2,0	289,13	2,0	110,13
**30**	6,2	54,50	6,2	26,62	4,9	26,62	4,9	126,13	2,0	387,73	2,0	149,24
**50**	6,2	22,23	6,3	8,96	4,8	8,96	4,8	116,52	2,0	412,82	2,0	160,37

[a)] arbitrary units; cf. Figure 5

3rd determination [a)]

ΔCV [V]	RNAse S 1-19..21-124		RNAse S 1-20..22-124		S-Protein 21-124		S-Protein 22-124		S-Peptide 1-19		S-Peptide 1-20	
	$m_{(1)}+$	$h_{(1)}$	$m_{(2)}+$	$h_{(2)}$	$p_{(1)}+$	$j_{(1)}$	$p_{(2)}+$	$j_{(2)}$	$n_{(1)}+$	$i_{(1)}$	$n_{(2)}+$	$i_{(2)}$
**3**	6,6	216,06	6,6	121,18	5,5	35,24	5,0	35,24	2,0	0,22	-	-
**8**	6,6	286,31	6,6	161,90	5,4	15,84	5,0	40,20	2,0	6,60	2,0	1,07
**11**	6,5	277,28	6,5	158,20	5,1	92,77	5,0	76,20	2,0	73,16	2,0	18,94
**13**	6,4	200,68	6,4	120,32	5,0	185,14	5,0	125,68	2,0	164,68	2,0	48,58
**15**	6,3	232,78	6,2	138,67	5,0	289,83	5,0	190,07	2,0	93,15	2,0	93,15
**17**	6,2	181,59	6,2	108,78	5,0	260,73	5,0	175,44	2,0	277,80	2,0	102,92
**20**	6,2	270,91	6,2	162,23	5,0	433,17	5,0	281,03	2,0	504,90	2,0	195,48
**30**	6,2	53,61	6,2	26,36	4,9	229,49	4,9	141,04	2,0	380,94	2,0	148,21
**50**	6,3	14,93	6,4	6,55	4,9	157,36	4,8	100,07	2,0	369,42	2,0	143,90

[a)] arbitrary units; cf. Figure 5

Supplemental Table 5: Ion intensities, charge states, and m/z values for FLAG-peptide - antiFLAG antibody complex at various collision cell voltage difference settings.

1st determination

Antibody + 2 peptides

**ion**	**m/z**	**20**	**40**	**60**	**80**	**100**	**120**	**150**	**170**	**200**
**21+**	7183	0,3	0,19	0,2	0	0	0	0	0	0
**22+**	6859	1	1	0,9	0,18	0,13	0	0	0	0
**23+**	6562	1,76	1,24	1,17	0,29	0,31	0,14	0	0	0
**24+**	6283	2,1	2,01	1,76	0,52	0,51	0,23	0	0	0
**25+**	6032	2,94	3,15	2,52	0,65	0,51	0,34	0	0	0
**26+**	5797	1,89	1,84	1,43	0,36	0,42	0,2	0	0	0
**27+**	5586	0,6	0,52	0,3	0	0	0	0	0	0

Antibody + 1 peptide

**ion**	**m/z**	**20**	**40**	**60**	**80**	**100**	**120**	**150**	**170**	**200**
**21+**	7149	0,14	0,1	0,36	0	0	0	0	0	0
**22+**	6814	0,54	1,12	0,78	0,34	0,29	0	0	0	0
**23+**	6516	0,74	1,73	1,31	0,56	0,4	0,21	0	0	0
**24+**	6239	0,78	2,44	1,75	0,6	0,78	0,26	0	0	0
**25+**	5993	0,65	2,58	2,26	0,76	0,61	0,44	0	0	0
**26+**	5761	0,3	1,74	1,47	0,56	0,57	0	0	0	0
**27+**	5542	0,12	0,2	0,5	0,2	0,16	0	0	0	0

Antibody

**ion**	**m/z**	**20**	**40**	**60**	**80**	**100**	**120**	**150**	**170**	**200**
**21+**	7094	0	0	0,2	0	0	0	0	0	0
**22+**	6767	0	0,5	0,49	0,17	0,18	0	0	0	0
**23+**	6472	0	0,67	0,7	0,26	0,18	0,15	0	0	0
**24+**	6199	0	0,77	0,68	0,23	0,27	0,23	0	0	0
**25+**	5949	0	0,86	0,68	0,32	0,26	0,16	0	0	0
**26+**	5721	0	0,55	0,53	0,19	0,16	0,15	0	0	0
**27+**	5513	0	0	0	0	0	0	0	0	0

FLAG peptide

**ion**	**m/z**	**20**	**40**	**60**	**80**	**100**	**120**	**150**	**170**	**200**
**1+T**	3037	0	0	0	0	0	0	0	0	0
**1+D**	2025	0	0	0	0	0	0	0	0	0
**1+**	1013	0	0	0	0,91	3,86	6,15	5,73	4,22	3,61
**2+**	507	0	0	0	1,14	2,96	2,12	2,35	1,82	1,14
**3+**	338	0	0	0	0	0	0	0	0	0

(T=trimer; D=dimer)

Supplemental Table 5: continued

2nd determination

Antibody + 2 peptides

ion	m/z	20	40	60	70	80	100	150	170	200
21+	7183	0,44	0,62	0,88	0,53	0,58	0	0	0	0
22+	6859	1	1,98	2,37	2,53	1,69	1	0,3	0	0
23+	6562	1,27	3,4	5,07	4,49	3,4	1,76	0,81	0	0
24+	6283	1,86	5,86	10,5	7,35	5,23	3,17	1,29	0	0
25+	6032	2,17	7,8	12	10,7	6,92	4,25	1,97	0	0
26+	5797	1	4,08	5,47	4,35	3,35	2,44	1,67	0	0
27+	5586	0,25	0,85	0,76	0,76	0,61	0,5	0,42	0	0

Antibody + 1 peptide

ion	m/z	20	40	60	70	80	100	150	170	200
21+	7149	0,44	0,78	1,19	1,13	0,88	0	0	0	0
22+	6814	1,25	2,69	3,62	3,41	2,96	1,55	0,59	0	0
23+	6516	1,58	4,22	6,81	6,39	5,18	3,14	1,14	0	0
24+	6239	1,86	5,97	10,4	8,72	7,58	4,45	2,33	0	0
25+	5993	1,89	7,39	10,9	9,56	8,03	5,73	2,83	0	0
26+	5761	1,03	3,98	5,83	4,95	3,4	2,74	1,82	0	0
27+	5542	0,2	0,94	1,06	0,92	0,85	0,55	0,45	0	0

Antibody

ion	m/z	20	40	60	70	80	100	150	170	200
21+	7094	0	0,49	0,57	0,45	0,51	0	0	0	0
22+	6767	0	1,14	1,91	1,66	1,28	0,75	0,3	0	0
23+	6472	0	1,63	2,65	2,63	2,05	1,16	0,63	0	0
24+	6199	0,62	1,71	3,11	2,48	2,43	1,69	0,85	0	0
25+	5949	0,53	2,02	2,81	2,33	1,81	1,53	1,01	0	0
26+	5721	0,38	1,17	1,49	1,58	0,97	0,93	0,58	0	0
27+	5513	0	0,37	0,37	0,39	0,33	0	0	0	0

FLAG peptide

ion	m/z	20	40	60	70	80	100	150	170	200
1+T	3037	0	0	0	0	0	0	0	0	0
1+D	2025	0	0	0	0	0	0	0	0	0
1+	1013	0	0	1,18	7,03	8,75	12,5	3,15	3,15	3,15
2+	507	0	0	3,32	4,77	8	4,68	0	0	0
3+	338	0	0	0	0	0	0	0	0	0

(T=trimer; D=dimer)

Supplemental Table 5: continued

3rd determination

Antibody + 2 peptides

ion	m/z	40	60	70	80	100	120	150	170
21+	7183	0	0	0	0	0	0	0	
22+	6859	0,79	0,63	0,61	0,39	0,51	0,1	0	0
23+	6562	1,63	1,39	1,24	1,31	1,02	0,3	0	0
24+	6283	3,45	3,19	3,33	2,62	2,05	0,84	0	0
25+	6032	5,98	5,36	5,94	4,77	3,35	1,81	0,47	0
26+	5797	4,44	4,36	4	3,57	2,39	1,33	0,39	0
27+	5586	1,35	1,17	1,12	0,89	0,7	0,61	0	0

Antibody + 1 peptide

ion	m/z	40	60	70	80	100	120	150	170
21+	7149	0	0	0	0	0	0	0	
22+	6814	0	0,63	0,64	0,65	0,52	0,32	0	0
23+	6516	0	1,53	1,97	1,82	1,45	0,67	0,2	0
24+	6239	0	2,44	3,24	3,2	2,43	1,77	0,34	0
25+	5993	0	4,3	5,43	4,84	3,53	1,97	0,5	0
26+	5761	0	3,41	3,73	3,87	3	1,59	0,39	0
27+	5542	0	0,1	1,3	1,09	0,99	0,5	0	0

Antibody

ion	m/z	40	60	70	80	100	120	150	170
21+	7094	0	0	0	0	0	0	0	0
22+	6767	0	0,29	0,32	0,39	0,38	0	0	0
23+	6472	0	0,51	0,51	0,87	0,64	0,29	0	0
24+	6199	0	0,86	0,99	1,23	0,83	0,54	0,21	0
25+	5949	0	1,09	1,58	1,49	1,13	0,75	0,33	0
26+	5721	0	1,13	1,27	0,88	0,76	0,44	0,24	0
27+	5513	0	0,35	0,52	0,45	0	0	0	0

FLAG peptide

ion	m/z	40	60	70	80	100	120	150	170
1+T	3037	0	0	0	0	0	0	0	0
1+D	2025	0	0	0	0	0	0	0	0
1+	1013	0	0	1,71	4,97	7,44	5,97	1,36	1,36
2+	507	0	0	2,28	3,75	4,51	1,9	0	0
3+	338	0	0	0	0	0	0	0	0

(T=trimer; D=dimer)

Supplemental Table 6: Apex heights and mean charge states of educt and product ion signals upon gas phase dissociation of FLAG-peptide – antiFLAG antibody complex.

**ΔCV [V]**	**1st determination a)**								**2nd determination a)**								**3rd determination a)**							
	$m_{(1)}$+	$h_{(1)}$	$m_{(2)}$+	$h_{(2)}$	n+	i	p+	j	$m_{(1)}$+	$h_{(1)}$	$m_{(2)}$+	$h_{(2)}$	n+	i	p+	j	$m_{(1)}$+	$h_{(1)}$	$m_{(2)}$+	$h_{(2)}$	n+	i	p+	j
**4**	23,8	0,804	24,4	2,913	-	0	-	0	-	0	24	1,868	-	0	-	0	-	0	-	5,981	-	0	-	0
**20**	23,8	0,804	24,4	2,913	-	0	-	0	23,9	1,871	24	2,137	-	0	-	0	-	0	-	5,981	-	0	-	0
**40**	24,2	2,611	24,5	3,134	-	0	24,1	0,834	24,2	7,032	24,4	7,668	-	0	24	1,199	-	0	24,9	5,981	-	0	-	0
**60**	24,3	2,145	24,3	2,455	-	0	23,8	0,748	24,2	11,364	24,3	12,551	1,7	3,534	23,9	2,421	24,7	4,454	24,9	5,492	-	0	24,8	1,142
**70**	n.d.	n.d.	n.d.	n.d.	n.d.	n.d.	n.d.	n.d.	24,2	9,666	24,3	10,529	1,4	7,248	23,9	1,979	24,8	5,142	24,9	5,933	1,6	2,172	24,9	1,612
**80**	24,4	0,704	24,4	0,645	1,6	1,217	24,1	0,337	24,1	8,254	24,3	6,657	1,5	9,165	23,9	1,765	24,8	4,776	24,9	4,798	1,4	5,147	24,6	1,413
**100**	24,5	0,726	24,4	0,56	1,4	4,003	24	0,293	24,4	5,345	24,5	4,129	1,3	12,28	24,1	1,016	24,8	3,49	24,8	3,314	1,4	7,644	24,3	1,044
**120**	24,2	0,401	24,7	0,328	1,3	6,059	24,4	0,226	24,6	2,843	24,8	1,958	1,2	9,043	24,3	0,324	24,8	2,087	25,2	1,81	1,3	6,019	24,7	0,743
**150**	-	0	-	0	1,3	5,609	-	0	-	0	-	0	1	3,199	-	0	24,8	0,503	25,5	0,482	1	1,381	25	0,352
**170**	-	0	-	0	1,3	4,308	-	0	-	0	-	0	1	3,199	-	0	-	0	-	0	1	1,381	-	0
**200**	-	0	-	0	1,2	3,64	-	0	-	0	-	0	1	3,199	-	0	-	0	-	0	1	1,381	-	0

a) arbitrary units; cf. Figure 6; n.d.: not determined

Supplemental Table 7: Ion intensities, charge states, and m/z values for TroponinI peptide - antiTroponinI at various collision cell voltage difference settings.

1st determination

Troponin I peptide

ion	m/z	2V	4V	6V	8V	12V	16V	20V	30V	40V	50V	60V	70V	80V
D1+	3629	10	10	10	10	10	10	10	10	10	10	10	10	10
1+	1815	6	8	7	7	12	11	12	26	45	63	111	133	152
2+	908	118	187	256	352	692	1634	2155	4871	6635	9454	11135	10995	11326
3+	606	59	76	126	134	238	513	692	1254	1788	2324	2294	2234	2118
4+	454	10	10	10	10	10	10	10	10	10	10	10	10	10

antibody

ion	m/z	2V	4V	6V	8V	12V	16V	20V	30V	40V	50V	60V	70V	80V
28+	5231	506	582	542	400	330	773	652	643	588	587	581	513	507
27+	5424	1963	2302	2188	1773	1616	2834	2646	2631	2519	2622	2564	2315	2218
26+	5632	3689	4083	4114	3696	3484	5073	4974	5110	5317	5485	5376	5124	5021
25+	5860	2851	3293	3291	3274	3274	3893	3959	4544	4877	5184	5348	4993	5039
24+	6102	1315	1404	1517	1659	1685	1712	1835	2330	2667	2971	3142	3031	3131
23+	6365	381	363	404	494	551	496	582	811	1124	1175	1339	1303	1359

antibody with 1 peptide

ion	m/z	2V	4V	6V	8V	12V	16V	20V	30V	40V	50V	60V	70V	80V
28+	5296	1185	1512	1400	1025	872	1888	1667	1564	1413	1381	1295	1102	1024
27+	5489	4192	4916	4812	4034	3773	6061	5581	5683	5456	5356	5065	4633	4530
26+	5701	6474	7368	7391	7017	6850	8697	8623	9133	9295	9384	9198	8567	8360
25+	5930	4458	4767	5074	5132	5196	5764	5828	6548	7029	7181	7353	6989	7002
24+	6179	1770	1816	2020	2231	2283	2206	2417	3059	3366	3591	3768	3550	3697
23+	6446	388	419	445	524	597	533	608	859	1062	1168	1314	1270	1312

antibody with 2 peptides

ion	m/z	2V	4V	6V	8V	12V	16V	20V	30V	40V	50V	60V	70V	80V
28+	5359	726	901	842	632	528	1128	1022	968	789	741	669	603	533
27+	5558	2275	2588	2607	2238	2072	3214	2949	2894	2716	2540	2413	2204	2107
26+	5773	3056	3320	3392	3349	3322	3993	3846	3911	4042	3822	3681	3425	3421
25+	6003	1805	1916	2045	2150	2104	2302	2259	2484	2581	2505	2486	2306	2307
24+	6250	618	638	688	753	822	748	800	892	1021	957	1020	905	967
23+	6523	121	129	150	156	167	156	171	195	239	254	271	262	286

D = dimer; ion intensity values were imputed
4+ ion intensity values were imputed

Supplemental Table 7: continued

2nd determination

Troponin I peptide

ion	m/z	2V	4V	6V	8V	12V	16V	20V	30V	40V	50V	60V	70V	80V
D1+	3629	10	10	10	10	10	10	10	10	10	10	10	10	10
1+	1816	5	5	8	8	7	16	8	18	29	58	94	135	156
2+	908	75	111	193	328	465	1052	1709	3463	6093	7789	10328	10711	10635
3+	606	31	52	91	121	153	289	448	799	1373	1513	1621	1573	1376
4+	455	10	10	10	10	10	10	10	10	10	10	10	10	10

antibody

ion	m/z	2V	4V	6V	8V	12V	16V	20V	30V	40V	50V	60V	70V	80V
28+	5231	278	252	294	367	276	377	469	542	636	434	530	522	484
27+	5424	1222	991	1211	1536	897	1356	1608	1870	2164	1790	2002	1957	1785
26+	5632	2507	2038	2481	3083	1928	2504	3042	3327	3720	3465	3906	3782	3400
25+	5860	2259	1846	2289	2727	2017	2299	2567	2826	3119	3222	3550	3472	3149
24+	6102	1122	920	1119	1337	1242	1267	1352	1441	1603	1783	2020	2024	1860
23+	6365	308	262	349	422	418	432	434	526	590	732	812	840	759

antibody with 1 peptide

ion	m/z	2V	4V	6V	8V	12V	16V	20V	30V	40V	50V	60V	70V	80V
28+	5296	674	604	738	894	627	928	1165	1327	1550	1082	1212	1146	1018
27+	5489	2872	2334	2804	3500	1953	2980	3499	4040	4434	3693	3900	3787	3376
26+	5701	5092	3991	4896	6006	3268	4375	5216	5795	6203	5923	6374	6107	5356
25+	5930	3733	2981	3640	4547	2533	3097	3505	3807	4196	4523	4931	4606	4209
24+	6179	1559	1242	1547	1936	1205	1331	1496	1640	1857	2175	2405	2299	2129
23+	6446	364	310	400	471	341	382	410	492	566	710	807	790	754

antibody with 2 peptides

ion	m/z	2V	4V	6V	8V	12V	16V	20V	30V	40V	50V	60V	70V	80V
28+	5359	422	445	518	578	559	736	878	934	956	689	671	707	581
27+	5558	1615	1565	1749	2001	1904	2238	2529	2630	2578	2074	2017	1906	1759
26+	5773	2469	2316	2578	3025	2951	3135	3353	3329	3259	2959	2859	2693	2570
25+	6003	1531	1501	1647	2011	2124	2114	2171	2136	2102	2029	1990	1825	1828
24+	6250	545	508	615	729	857	810	853	808	807	903	867	789	848
23+	6523	112	114	128	143	191	189	193	196	235	256	263	268	304

D = dimer; ion intensity values were imputed
4+ ion intensity values were imputed

Supplemental Table 8: Apex heights and mean charge states of educt and product ion signals upon gas phase dissociation of TroponinI immune complex.

ΔCV [V]	1st determination a)								2nd determination a)							
	$m_{(1)}+$	$h_{(1)}$	$m_{(2)}+$	$h_{(2)}$	n+	i	p+	j	$m_{(1)}+$	$h_{(1)}$	$m_{(2)}+$	$h_{(2)}$	n+	i	p+	j
**2**	25,9	6370	26,1	3029	2,2	108	25,7	3611	25,7	4991	25,9	2390	2,2	79	25,5	2577
**4**	25,9	7203	26,1	3322	2,3	175	25,7	4107	25,7	3921	25,9	2260	2,3	130	25,5	2085
**6**	25,9	7271	26,1	3395	2,2	239	25,6	4094	25,7	4789	25,9	2502	2,2	220	25,5	2563
**8**	25,8	6881	26,0	3287	2,1	361	25,5	3794	25,7	5936	25,9	2957	2,1	332	25,5	3133
**12**	25,7	6756	25,9	3210	2,3	1186	25,4	3667	25,7	3192	25,8	2891	2,2	593	25,2	2126
**16**	26,0	8592	26,1	4041	2,2	2082	25,7	4990	25,8	4279	25,9	3082	2,2	1127	25,4	2575
**20**	25,9	8402	26,1	3825	2,2	2872	25,7	4917	25,9	5041	26,0	3310	2,1	1794	25,5	3014
**30**	25,8	8892	26,0	3886	2,1	5105	25,5	5237	25,9	5605	26,0	3322	2,0	3494	25,5	3300
**40**	25,7	9092	26,0	3931	2,2	7176	25,4	5469	25,9	6036	26,0	3235	2,1	6230	25,6	3686
**50**	25,7	9160	25,9	3721	2,1	9712	25,4	5774	25,7	5795	25,9	2861	2,0	7907	25,4	3570
**60**	25,6	9057	25,9	3580	2,0	11192	25,3	5816	25,7	6231	25,9	2773	2,0	10191	25,4	3984
**70**	25,6	8495	25,9	3323	2,0	11050	25,3	5485	25,7	5920	25,9	2582	2,0	10785	25,4	3878
**80**	25,6	8389	25,9	3291	1,9	11501	25,3	5482	25,7	5261	25,8	2460	2,1	10900	25,4	3508

a) arbitrary units; cf. Figure 7
